# Single-cell transcriptomes reveal cell-type-specific and sample-specific gene function in human cancer

**DOI:** 10.1016/j.heliyon.2025.e42218

**Published:** 2025-01-23

**Authors:** Huating Yuan, Xin Liang, Xinxin Zhang, Yu Cao

**Affiliations:** a College of Biology and Engineering, Guizhou Medical University, Guiyang, China; b College of Bioinformatics Science and Technology, Harbin Medical University, Harbin, China; c Institute of Big Health, Guizhou Medical University, Guiyang, China

**Keywords:** Single-cell RNA-Sequencing, Cancer, Gene function, Functional heterogeneity, Sample specificity, Cell-type specificity

## Abstract

Accurate annotation of gene function in individual samples and even in each cell type is essential for understanding the pathogenesis of cancers. Single-cell RNA-sequencing (scRNA-seq) provides unprecedented resolution to decipher gene function. In order to explore how scRNA-seq contributes to the understanding of gene function in cancers, we constructed an assessment framework based on co-expression network and neighbor-voting method using 116,814 cells. Compared with bulk transcriptome, scRNA-seq recalled more experimentally verified gene functions. Surprisingly, scRNA-seq revealed cell-type-specific functions, especially in immune cells, whose expression profile recalled immune-related functions that were not discovered in cancer cells. Furthermore, scRNA-seq discovered sample-specific functions, highlighting that it provided sample-specific information. We also explored factors affecting the performance of gene function prediction. We found that 500 or more cells should be considered in the prediction with scRNA-seq, and that scRNA-seq datasets generated from 10x Genomics platform had a better performance than those from Smart-seq2. Collectively, we compared the prediction performance of bulk data and scRNA-seq data from multiple perspectives, revealing the irreplaceable role of single-cell sequencing in decoding the biological progresses in which the gene involved.

## Graphical abstract

**Figure:**
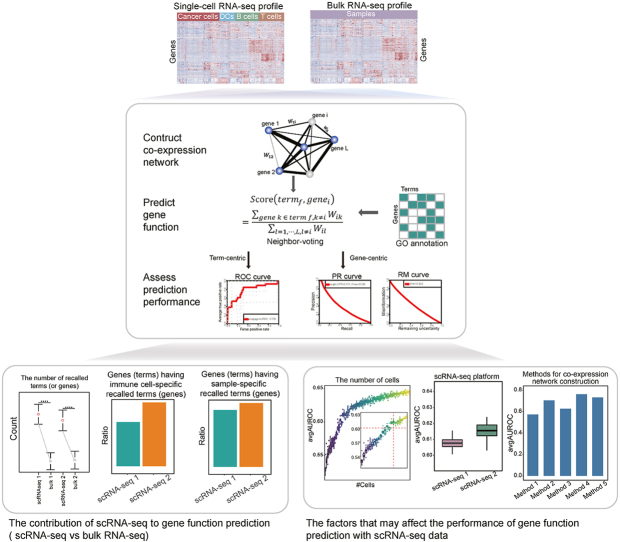

## 1 Introduction

Accurate annotation of gene function is key to understanding the pathogenesis of complex diseases, including cancers, at the molecular level, and has important biomedical and pharmaceutical significance. Low-throughput biological experiments can often provide rich and detailed information about gene function, but limited by time and cost. Therefore, computational annotation of gene function based on high-throughput sequencing data has been widely explored. Since genes with similar expression patterns were thought to be involved in the same cellular pathway or function [[1], [2], [3], [4], [5]], RNA-seq data has been one of the most commonly used data types in inferring gene function. However, bulk RNA-seq measures the average expression level across all cells in a sample, masking the difference between cells, especially in cancers, which have extensive intra-tumoral heterogeneity. Thus, it is necessary to explore the gene function in cancers at a higher resolution.

Single-cell RNA-sequencing has revolutionized tumor research, and provided unprecedented resolution to decipher gene function [6]. Profiling 5902 single cells from 18 head and neck cancer samples by scRNA-seq, Puram et al. found that laminin *LAMC2* was involved in EMT, epithelial differentiation and cell cycle in 3 different samples, respectively, and that major histocompatibility complex *HLA-DRA* was associated with stress, epithelial differentiation, cell cycle and MHC class II protein complex in 4 different samples, respectively [7]. Furthermore, *TGFBI* has been reported to participate in the p-EMT process of tumor cells [7], at the same time, it was a marker of M2 macrophages, and also can regulate the activity of T cells [8]. Therefore, annotating gene function in cancer with scRNA-seq data may contribute to the understanding of the functional heterogeneity of genes, so as to understand the pathological mechanism of cancers.

In this study, we constructed a function prediction framework based on co-expression network and neighbor-voting method to systematically assess the contribution of scRNA-seq to understanding gene function in cancer. By comparing the results between scRNA-seq data and bulk RNA-seq data, we found that single-cell datasets recalled more functions than bulk datasets. Furthermore, we predicted gene functions in individual samples and different cell types, the results highlighted the unique value of scRNA-seq in understanding gene function. Finally, we explored the factors that may affect the performance of gene function prediction with scRNA-seq, so as to give some suggestions to the follow-up studies.

## 2 Materials and methods

### 2.1 Processing single-cell RNA-seq data and TCGA RNA-seq data

The scRNA-seq data was downloaded from the Gene Expression Omnibus (GEO) database, ArrayExpress database or Genome Sequence Archive (GSA) database [9,10]. The cell type identities were taken from the corresponding studies [7,[11], [12], [13], [14], [15], [16], [17], [18], [19]]. The expression value was first converted to transcripts-per-million (TPM), and then quantified as described in Equation (E1).

(E1) $$E_{i,j}=\log_{2}\left(\frac{{TPM}_{i,j}}{10}+1\right)\text{,}$$

where ${TPM}_{i,j}$ refers to the TPM value of gene i (i=1,⋯,L,L is the number of genes in the single-cell expression profile) in cell j (j=1,⋯,N, N is the number of cells in the single-cell expression profile). The TPM values were divided by 10 because the complexity of the single-cell library was estimated to be on the order of 100,000 transcripts. TPM will count each transcript ∼10 times, which may inflate the differences between positive and zero TPM values [7,11,20]. Considering the low sensitivity and high technical noise of scRNA-seq assay, we carried out two steps for quality control. Firstly, we calculated two quality metrics for each cell, including the number of genes with detectable expression ($E_{i,j}>0$) and the average expression level of 95 housekeeping genes from Ref. [20]. We then excluded cells with the number of expressed genes less than 2000 or with the average housekeeping expression below 2.5. Secondly, for the remaining cells, we calculated the aggregate expression of gene i using Equation (E2), as well as the proportion of cells in which the gene was detected. Then, genes with $E_{a}>2$ and were detected in at least 20 % of cells were retained (Table S1).

(E2) $$E_{a}\left(i\right)=\log_{2}\left(\frac{\sum_{j=1}^{N}{TPM}_{i,j}}{N}+1\right)\text{,}$$

The bulk RNA-seq data (TPM values) was downloaded from TCGA (https://portal.gdc.cancer.gov/). Expression levels were quantified using Equation (E3).

(E3) $$E_{TCGA}=\log_{2}\left(TPM+1\right)\text{,}$$

samples with less than 2000 expressed genes ($E_{TCGA}>0$) were discarded, and genes with detectable expression in at least 20 % of samples were retained (Table S2).

### 2.2 GO annotations and tested genes

We obtained gene function annotations from the “org.Hs.egGO2ALLEGS” (R package “org.Hs.eg.db”) [21] object which provides mappings between a GO term and all of the genes annotated to it or to one of its child nodes in the GO ontology, and only the Biological Process (BP) terms were used here. The relationship between a gene and a BP term was considered experimentally verified if it had EXP, IDA, IMP, IGI, IEP, TAS or IC evidence codes. All experimentally verified relationships, including 14,545 GO BP terms and 15,137 genes, were selected as the gold standard. The tested genes were defined as follows: 1) annotated with at least two GO BP terms, 2) detected both in single-cell and bulk expression profiles.

### 2.3 Constructing single-cell co-expression network

The single-cell co-expression network was constructed by calculating the correlation coefficients between all genes based on the single-cell expression profile. The correlation coefficients were ranked and normalized to between 0 and 1, and then were defined as the weights of edges, so as to reduce the effects of changes in the actual correlation distribution (induced by outlier samples, etc.) on the results [22]. The degree of a node was calculated as the total weights of all edges connected to it [22] (Fig. S1).

In order to test the effects of different co-expression metrics on gene function prediction, we used Spearman rank correlation coefficients, Pearson correlation coefficients and mutual information (MI) to construct co-expression networks. The MI was computed using “build.mim” function in R package “minet” [23]. The effects of dropout events were tested by constructing two types of networks, with or without 0. The co-expression of two genes were calculated in all cells or only in cells with detectable expression of them to build networks with and without 0, respectively. Moreover, we also explored the influence of the negative correlation by using the correlation coefficients themselves or their absolute values to rank edges. There were 10 types of single-cell co-expression networks in total: networks constructed based on MI, Pearson or Spearman correlation coefficient, and without other processing (MI vs pearson vs spearman); networks constructed based on the three metrics and without 0 (MI_dp vs pearson_dp vs spearman_dp); networks whose edges were ranked by the absolute values of correlation coefficients (pearson_abs vs spearman_abs); networks constructed based on expression profile without 0 and the edges were ranked by the absolute values of correlation coefficients (pearson_dp_abs vs spearman_dp_abs).

The integrated network was generated by averaging the weights of corresponding edges across individual networks, then re-ranking and normalizing values to between 0 and 1. In order to compare with individual single-cell networks, we built 4 integrated single-cell networks: network integrated from the 2 MI networks (integrated_MI), from the 4 Pearson networks (integrated_pearson), from the 4 Spearman networks (integrated_spearman) and from all the 10 networks described above (integrated_all).

### 2.4 Neighbor-voting method

The functional relevance between genes and GO BP terms was measured using a simple but effective neighbor-voting algorithm, where a gene was given the labels of other genes in its neighborhood in the co-expression network (Fig. S1). In this study, the score of the functional relevance between a gene and a BP term was defined as the ratio of the total weights of the gene's neighbors associated with this term to the degree of this gene [[24], [25], [26]], and was calculated as described in Equation (E4).

(E4) $$Score\left({term}_{f},{gene}_{i}\right)=\frac{\sum_{{gene}_{k}\in {term}_{f,}\;k\neq i}W_{ik}}{\sum_{l=1,\cdots ,L,l\neq i}W_{il}}\text{,}$$

where f is a GO BP term, $W_{ik}$ ($W_{il}$) is the weight of edge between gene i and gene k (gene l) in the co-expression network. A gene was predicted to relate to a term when the relevance score was greater than or equal to a given threshold. For a tested gene, terms in the intersection between its predicted terms and its experimentally validated terms, in other words, the truly positive terms, were defined as its recalled terms. For a term, genes in the intersection between its predicted genes and its experimentally validated genes, that is, the truly positive genes, were defined as its recalled genes.

### 2.5 Evaluation metrics

Two modes of evaluations were used here: gene-centric and term-centric [[27], [28], [29]] (Fig. S1).

The gene-centric evaluation measures how accurately the functional terms can be assigned to a gene. The precision–recall (PR) curves and remaining uncertainty–misinformation (RU-MI) curves were used as the two chief metrics here.

In general, for a given gene, the positive terms (terms associated with this gene) account for a small part of all the terms, resulting that the negative terms are much more than the positive ones. Therefore, the PR curve, which only calculates the positive instances (including the true positives, the false positives and the false negatives) rather than the negative ones (the true negatives), is the most suitable in this situation. And simultaneously, it has high interpretability. For a tested gene i, the precision (${\mathrm{pr}}_{i}\left(t\right)$) and recall (${rc}_{i}\left(t\right)$) were calculated according to Equations (E5), (E6), (E7).

(E5) $${\mathrm{pr}}_{i}\left(t\right)=\frac{\sum_{f}I\left(f\in P_{i}\left(t\right)⋀f\in T_{i}\right)}{\sum_{f}I\left(f\in P_{i}\left(t\right)\right)}\text{,}$$

(E6) $${rc}_{i}\left(t\right)=\frac{\sum_{f}I\left(f\in P_{i}\left(t\right)⋀f\in T_{i}\right)}{\sum_{f}I\left(f\in T_{i}\right)}\text{,}$$

(E7) $$I\left(x\right)=\left\{ \begin{array}{c} 1,if\;x\;is\;TRUE\\ 0,else\; \end{array}\right.\text{,}$$

where t is the decision threshold and varies from 0.01 to 1.00 with a step size of 0.01. $T_{i}$ is a set of experimentally validated terms for gene i, and $P_{i}\left(t\right)$ is a set of predicted terms for gene i with the relevance score greater than or equal to t. I(x) is an indicator function. For a series of thresholds, a PR curve for gene i would be plotted, and the area under the PR curve (AUPRC) was calculated using the trapezoid rule.

In order to assess the overall performance, the performance of all genes needs to be integrated. Therefore, we defined the average PR curves. For a fixed threshold t, a point on the average PR curve was created by averaging precision and recall across all tested genes. The average precision (pr(t)) and recall (rc(t)) were calculated using Equations (E8), (E9).

(E8) $$\mathrm{pr}\left(t\right)=\frac{\sum_{i=1}^{m\left(t\right)}{\mathrm{pr}}_{i}\left(t\right)}{m\left(t\right)}\text{,}$$

(E9) $$rc\left(t\right)=\frac{\sum_{i=1}^{M}{rc}_{i}\left(t\right)}{M}\text{,}$$

where m(t) is the number of genes on which at least one prediction was made above threshold *t*, and M is the number of tested genes. The area under the average PR curve (avgAUPRC) was also calculated based on the trapezoid rule. And the $F_{\max}$ was computed as the harmonic mean between the average precision and recall (Equation (E10)).

(E10) $$F_{\max}=\max_{t}\left\{\frac{2\cdot \mathrm{pr}\left(t\right)\cdot rc\left(t\right)}{\mathrm{pr}\left(t\right)+rc\left(t\right)}\right\}$$

The value of AUPRC, avgAUPRC or $F_{\max}$ ranges from 0 to 1, and a higher value indicates a better performance.

The RU-MI curve measures the overall error level of gene function prediction. It incorporates the information content of each GO term besides counting the number of false positives, false negatives, etc., so as to reward the methods predicting more difficult or less frequent terms. The information content of term f was calculated using Equation (E11).

(E11) $$IC\left(f\right)=\log_{2}\frac{1}{\mathrm{Pr}\left(f\right)}\text{,}$$

where Pr(f) is the relative frequency that a randomly selected gene will be associated with term f in the BP ontology [27]. Therefore, the more genes a term contains, the less information content it has. The remaining uncertainty (ru), misinformation (mi), and the corresponding minimum semantic distance ($S_{\min}$) were calculated according to Equations (E12), (E13), (E14).

(E12) $$ru\left(t\right)=\frac{1}{M}\sum_{i=1}^{M}\frac{\sum_{f}IC\left(f\right)\cdot I\left(f\notin P_{i}\left(t\right)⋀f\in T_{i}\right)}{\sum_{f}IC\left(f\right)\cdot I\left(f\in P_{i}\left(t\right)⋁f\in T_{i}\right)}\text{,}$$

(E13) $$mi\left(t\right)=\frac{1}{M}\sum_{i=1}^{M}\frac{\sum_{f}IC\left(f\right)\cdot I\left(f\in P_{i}\left(t\right)⋀f\notin T_{i}\right)}{\sum_{f}IC\left(f\right)\cdot I\left(f\in P_{i}\left(t\right)⋁f\in T_{i}\right)}\text{,}$$

(E14) $$S_{\min}=\min_{t}\left\{\sqrt{{ru\left(t\right)}^{2}+{mi\left(t\right)}^{2}}\;\right\}\text{,}$$

The $S_{\min}$ ranges between 0 and 1, and a lower value indicates a better performance.

The term-centric evaluation is an example of binary classification models, in which a given term is assigned (or not) to an input gene. Here, the most widely used performance metric in binary classification, the area under the receiver operating characteristic curve (AUROC), was calculated using R package “ROCR” [30]. It was used to assess the probability that we would be right about classifying genes as belonging to a particular term or not. After performing 10-fold cross validation, we calculated the average AUROC for each term, and the mean value of all the average AUROCs (avgAUROC) was used to measure the overall performance. To more confidently assess the performance, only terms annotated with 20-1000 genes were considered, leaving 3895 terms finally.

### 2.6 Stepwise analysis for the number of single cells

In order to assess the effect of cell number on the performance of gene function prediction, we randomly selected n(n∈(1,N)) cells from the single-cell expression profile, and then calculated the prediction performance based on the new expression profile. The above process for each n value was repeated 10 times to obtain the average performance.

### 2.7 Statistical analysis

One-sided Wilcoxon signed rank test was used for paired comparisons between two groups. All of the statistical analyses were performed using R program 3.6.2 (http://www.r-project.org) and Bioconductor.

## 3 Results

### 3.1 Single-cell RNA-seq discovered more experimentally verified gene functions than bulk RNA-seq

Based on the generally accepted hypothesis that genes with similar expression patterns have a high probability of sharing similar functions [[1], [2], [3], [4], [5]], we constructed a framework for gene function prediction using co-expression network and neighbor-voting method (Fig. S1). The performance of this framework was evaluated by Precision–recall (PR) curves, remaining uncertainty–misinformation (RU-MI) curves and ROC curves.

**Fig. 1:**
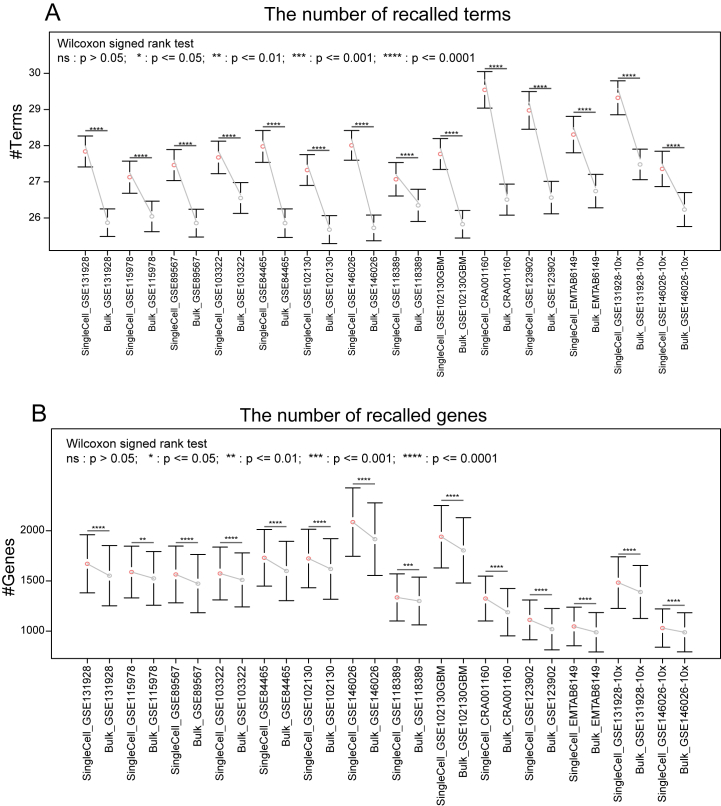
Single-cell RNA-seq recalled more gene functions than bulk RNA-seq. (A) The number of recalled terms for each tested gene in scRNA-seq or bulk RNA-seq. (B) The number of recalled genes for each term in scRNAs-seq or bulk RNA-seq.

**Fig. 2:**
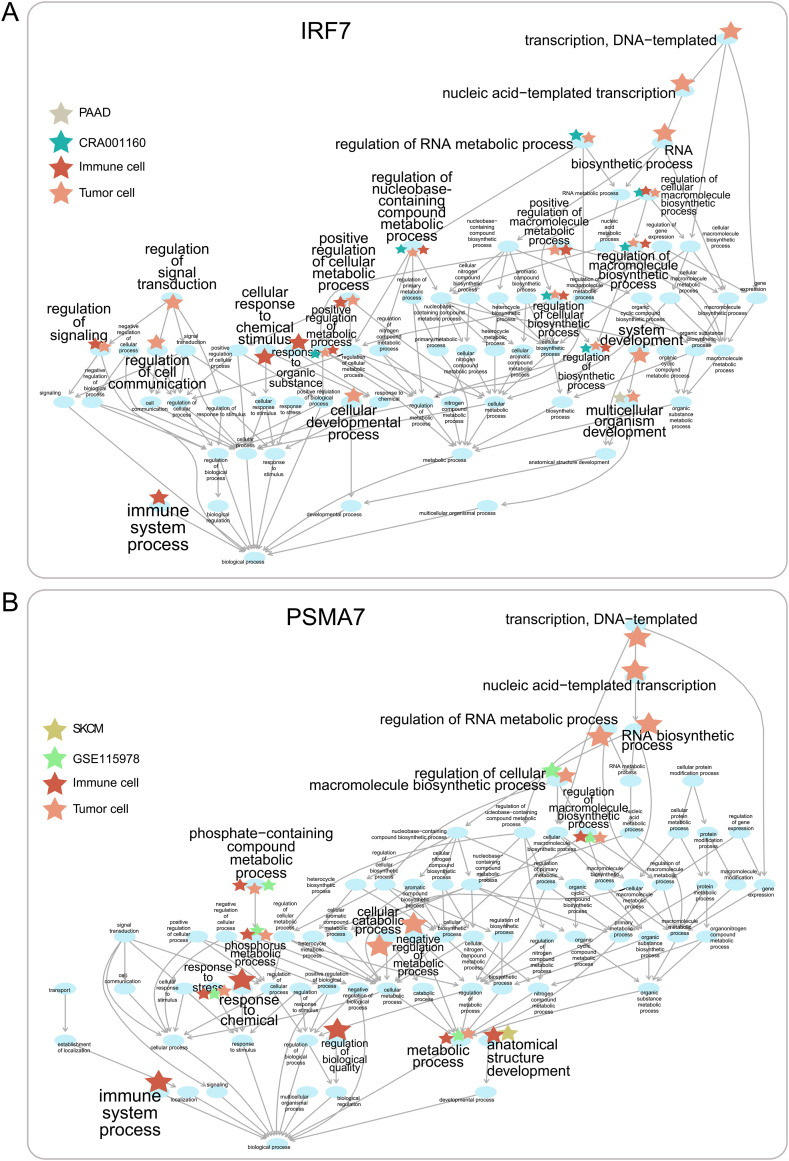
The directed acyclic graph (DAG) of recalled terms for tested genes. A term marked with an asterisk meant that it was discovered in the corresponding dataset, while a term not marked with asterisks meant that it was discovered in all the four datasets. The DAG of all the recalled terms for gene (A) *IRF7* and (B) *PAMA7*.

Traditionally, gene function prediction was mainly based on bulk data. Therefore, we first compared the overall performance between single-cell and bulk transcriptome expression data (Tables S1–2). In gene-centric evaluation, after performing leave-one-out cross validation, most single-cell datasets had higher avgAUPRC values, higher $F_{\max}$ scores and lower $S_{\min}$ values than the corresponding bulk datasets, but the difference was very slightly (Figs. S2A–C). Thus, the results of gene-centric evaluation showed that single-cell data had a comparable performance to the bulk data. Next, we focused on the performance for individual genes, and explored the difference in the recalled terms (the true positive terms) between single-cell and bulk data. We found that in all of the 14 single-cell datasets, most of the tested genes had a better performance in single-cell data (*P* < 0.05, Fig. S3A). Furthermore, we explored the difference in the recalled terms of each tested gene between the single-cell and bulk. The prediction threshold value was selected to correspond to the point in the average PR curve that provided the $F_{\max}$ score [27] (Table S3). After performing the neighbor-voting method to calculate the relevance scores between genes and terms, we found that scRNA-seq can discover much more experimentally validated terms (*P* < 0.05, Fig. 1A). For example, interferon regulatory factor 7 (*IRF7*), which has been widely reported to involve in immune response [31], viral defense [32], transcriptional regulation [31,33] and signal transduction [34], was correctly predicted to relate to 58 and 52 terms in scRNA-seq data CRA001160 [35] and the corresponding bulk data PAAD, respectively. And among these recalled terms, 51 terms were recalled both in CRA001160 and PAAD; 1 term “multicellular organism development” (GO:0007275) was recalled only in PAAD; while 7 terms, such as “regulation of RNA metabolic process” (GO:0051252) and “regulation of cellular macromolecule biosynthetic process” (GO:2000112), were only discovered in CRA001160 (Fig. 2A). Similar results were also observed for gene *PSMA7* (Fig. 2B). These results showed that more experimentally validated gene functions can be discovered when using scRNA-seq.

**Fig. 3:**
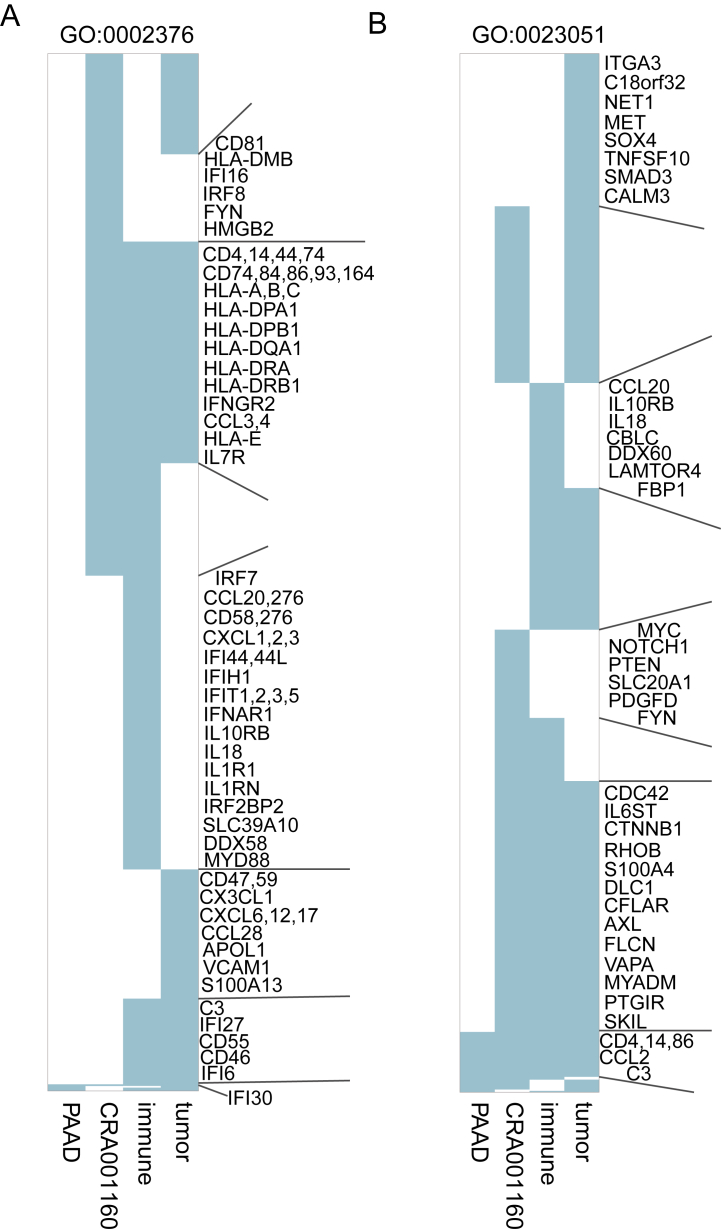
The recalled genes of individual terms in different datasets. A grid with light blue indicated that the gene was discovered in the corresponding dataset.

In term-centric evaluation, after performing 10-fold cross validation, the average AUROC for each term and avgAUROC across all terms were calculated. Although most of the single-cell datasets showed lower avgAUROC values than the corresponding bulk datasets, the difference was also very small. Moreover, the avgAUROC of CRA001160, which had the largest number of cells, was higher than that of the corresponding bulk data PAAD (Fig. S2D), suggesting that the avgAUROC of scRNA-seq data may be higher than that of the bulk data when increasing the cell number. Similarly, we explored the difference in the performance and the recalled genes (the true positive genes) for individual terms between single-cell and bulk data. It was found that although most terms had better performance in bulk data, the AUROC values in CRA001160 were significantly higher than those in PAAD (*P* < 0.05, Fig. S3B). Then, for a given term, we compared its recalled genes between single cell and bulk. Excitingly, more genes were recalled in scRNA-seq data than in bulk (*P* < 0.05, Fig. 1B). For example, for “immune system process” (GO:0002376), 312 and 4 experimentally verified genes were discovered in CRA001160 and PAAD, respectively. And among them, only one gene, *IFI30*, was discovered both in CRA001160 and PAAD; 3 genes only occurred in PAAD; while 311 genes, such as well-known immune-related genes *CD81*, *CD4* and *HLA-A*, were recalled in CRA001160 but not in PAAD (Fig. 3A). Similar phenomena were also observed for “regulation of signaling” (GO:0023051) (Fig. 3B). Taken together, more experimentally verified genes can be recalled with scRNA-seq.

### 3.2 Single-cell RNA-seq revealed gene functions related to the cell type

**Fig. 4:**
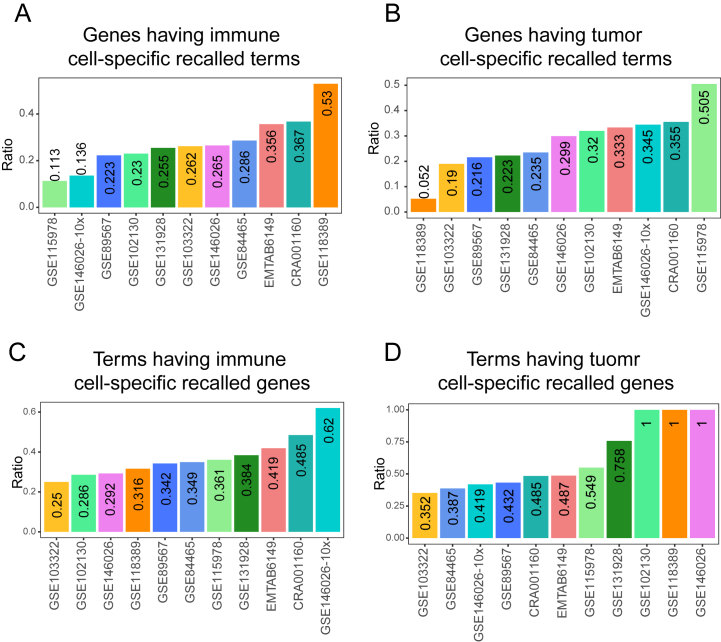
Single-cell RNA-seq revealed gene functions related to the cell type. (A) The ratio of genes having immune cell-specific recalled terms. (B) The ratio of genes having tumor cell-specific recalled terms. (C) The ratio of terms having immune cell-specific recalled genes. (D) The ratio of terms having tumor cell-specific recalled genes.

Single-cell sequencing enables us to distinguish different cell types from each other, so as to explore the roles of different cell types in the development of cancers. Tumor cells and immune cells are important components of tumor tissues, thus we planned to use scRNA-seq data of tumor cells and immune cells to predict gene functions. In gene-centric evaluation, the overall performance of bulk data, immune cell scRNA-seq data and tumor cell scRNA-seq data was comparable (Figs. S4A–C). The terms recalled by scRNA-seq data of immune cells and tumor cells were more than those recalled by bulk data (Figs. S5A–B). Furthermore, we compared the recalled terms of individual tested genes between immune cells and tumor cells from the same dataset. In 11 immune cell scRNA-seq datasets, an average of 27.49 % of the tested genes had at least one recalled term that was discovered in immune cells but not in tumor cells (Fig. 4A). Simultaneously, an average of 27.93 % of the tested genes had at least one recalled term that was discovered in tumor cells but not in immune cells (Fig. 4B). For example, with the scRNA-seq datasets of immune cells and tumor cells from CRA001160, a total of 72 experimentally verified terms were recalled for *IRF7*. Among these recalled terms, 3 terms, including “immune system process” (GO:0002376), were discovered only in immune cells, and 7 terms, such as “system development” (GO:0048731) and “regulation of cell communication” (GO:0010646), were recalled only in tumor cells (Fig. 2A). And similar results were obtained for *PSMA7* (Fig. 2B).

The term-centric evaluation showed a slightly lower performance of gene function prediction with both of the scRNA-seq profiles of immune cells and tumor cells (Fig. S4D), and again, both of the scRNA-seq profiles of immune cells and tumor cells recalled more genes than bulk data (Figs. S5C–D). Similarly, for each term, we compared its recalled genes between immune cells and tumor cells from the same dataset. On average, 37.31 % of the terms had at least one recalled gene discovered by scRNA-seq data of immune cells but not by that of tumor cells (Fig. 4C). And an average of 62.44 % of the terms had at least one recalled gene discovered by scRNA-seq data of tumor cells but not by that of immune cells, and particularly in three datasets (GSE102130 [13], GSE118389 [17] and GSE146026 [16]), the ratio reached 100 % (Fig. 4D). For instance, in the scRNA-seq datasets of immune cells and tumor cells from CRA001160, a total of 566 experimentally validated genes of “immune system process” (GO:0002376) were recalled, and among them, 242 recalled genes, such as *IRF7*, *CD58* and *IFNAR1,* were only discovered in immune cell scRNA-seq data; 138 recalled genes, such as *CD47*, *CX3CL1* and *CCL28*, were only discovered in tumor cell scRNA-seq data (Fig. 3A). And similar results were also observed for “regulation of signaling” (GO:0023051) (Fig. 3B).

Taken together, the results of both gene-centric and term-centric evaluation suggested that the single-cell expression profiles of different cell types can be used to predict gene functions related to the corresponding cell types.

### 3.3 Single-cell RNA-seq revealed sample-specific gene functions

**Fig. 5:**
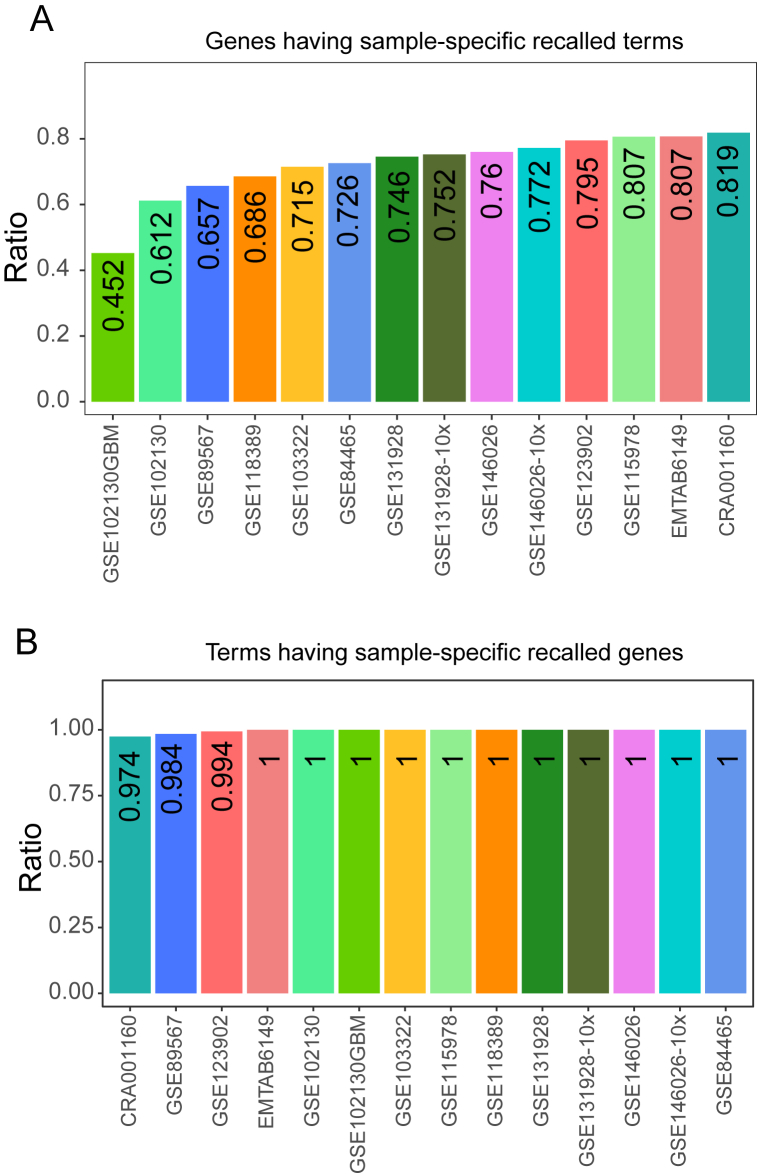
Single-cell RNA-seq revealed sample-specific functions. (A) The ratio of genes having sample-specific recalled terms. (B) The ratio of terms having sample-specific recalled genes.

The high resolution of single-cell sequencing enables us to explore cells within individual samples, which is expected to reveal the similarities and differences in the functions of a gene between different samples. Thus, we next explored the application of scRNA-seq in gene function prediction in individual samples. In gene-centric evaluation, the performance of the single-cell expression profiles of individual samples and the bulk expression profiles was also comparable (Figs. S6–8), and the recalled terms in single-cell data of individual samples were significantly more than those in bulk data (Figs. S9A–B). In order to explore the difference in gene functions between different samples, we compared the recalled terms between different samples for each tested gene. In each scRNA-seq dataset, we defined the terms which were recalled in some samples but not in all samples as the sample-specific recalled terms. On average, 72.19 % of tested genes had at least one sample-specific recalled term in the 14 scRNA-seq datasets (Fig. 5A). For example, in individual samples from CRA001160, 8 of the 21 recalled terms were sample-specific. Among them, “response to organic substance” (GO:0010033) was recalled in 2 samples; “cell surface receptor signaling pathway” (GO:0007166), “immune response” (GO:0006955) and “regulation of signal transduction” (GO:0009966) were recalled only in one sample (Fig. S10A). For individual samples from GSE115978 [15], 19 of the 60 recalled terms were sample-specific (Fig. S10B). These results suggested that single-cell expression profiles of individual samples can predict sample-specific gene functions.

In term-centric evaluation, a slightly lower performance was observed in the single-cell expression profiles of individual samples (Fig. S11), and the single-cell expression profiles of most individual samples recalled more genes (Figs. S9C–D). Similarly, for each term, we compared its recalled genes between different samples. In each scRNA-seq dataset, the recalled genes discovered in some samples but not in all samples were defined as sample-specific recalled genes. On average, 99.66 % of the terms had at least one sample-specific recalled gene in 14 scRNA-seq datasets, and especially in 11 datasets, the ratio reached 100 % (Fig. 5B). For instance, 75 experimentally validated genes of “positive regulation of response to stimulus” (GO:0048584) were recalled in individual samples from CRA001160, and all of them were sample-specific (Fig. S12A). Another example, in individual samples of GSE115978, all of the 130 experimentally verified genes of “intracellular signal transduction” (GO:0035556) were sample-specific (Fig. S12B).

Taken together, the results showed that for a gene, the single-cell expression profiles of individual samples can recall many sample-specific terms, and for a GO term, sample-specific genes also can be recalled by the single-cell expression profiles of individual samples, indicating the unique value of single-cell sequencing in gene function prediction and in revealing the functional heterogeneity between samples.

### 3.4 The impact of cell number on the performance of gene function prediction

The above systematically compared the gene function prediction performance between scRNA-seq data and bulk RNA-seq data from multiple perspectives, highlighting the irreplaceable value of single-cell sequencing in understanding gene function. Next, we will systematically explore the factors that may affect the performance of gene function prediction with scRNA-seq data.

**Fig. 6:**
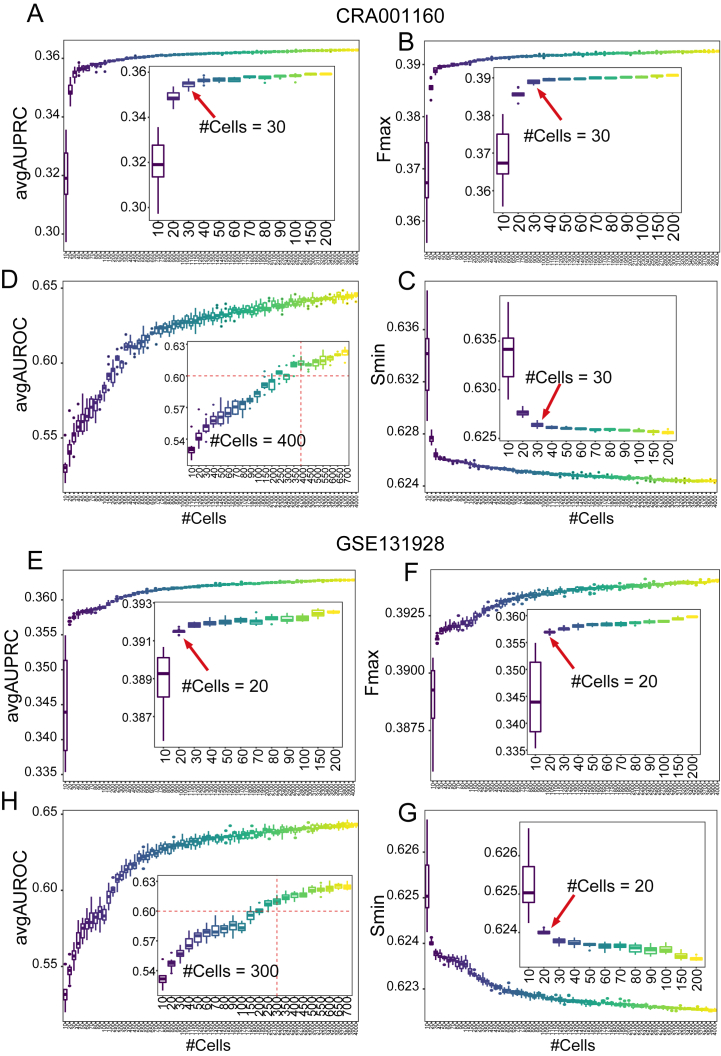
The impacts of cell number on the performance of gene function prediction. The association between the cell number and (A) avgAUPRC, (B) $F_{\max}$, (C) $S_{\min}$ and (D) avgAUROC in CRA001160. The association between cell number and (E) avgAUPRC, (F) $F_{\max}$, (G) $S_{\min}$ and (H) avgAUROC in GSE131928.

The number of cells is an important attribute of single-cell sequencing, thus, we planned to systematically assess the effects of cell number on the performance in a stepwise manner. In each step, a certain number of cells were randomly selected to construct a new expression profile, and the prediction performance was calculated. Each step was repeated 10 times to obtain the average performance. We found that the number of cells greatly influenced the performance of gene function prediction. In gene-centric evaluation, the avgAUPRC and $F_{\max}$ values improved as the number of cells was increased, and the $S_{\min}$ values gradually decreased with the increase of cell number, but the effect leveled off after a certain number of cells were used. In most of the scRNA-seq datasets, the effect of cell number on the prediction performance leveled off within 100 cells (Figs. S13–15). For example, the changes in performance tended to be stable after approximately 30 cells were used in CRA001160 (Fig. 6A–C), and this effect leveled off when using approximately 20 cells in GSE131928 [11] (Fig. 6E–G). From the view of term, the avgAUROC increased with the number of cells. In most datasets, the avgAUROC values of all the 10 repetitions were greater than 0.6 when approximately 500 cells were used (Fig. S16). For example, the avgAUROC values of all the 10 repetitions were greater than 0.6 when approximately 400 cells were used in CRA001160 (Fig. 6D), and for GSE131928, approximately 300 cells were required (Fig. 6H). In conclusion, the number of cells greatly affected the prediction performance, and 500 or more cells should be considered for gene function prediction with scRNA-seq.

### 3.5 The impact of single-cell sequencing platform on the performance

**Fig. 7:**
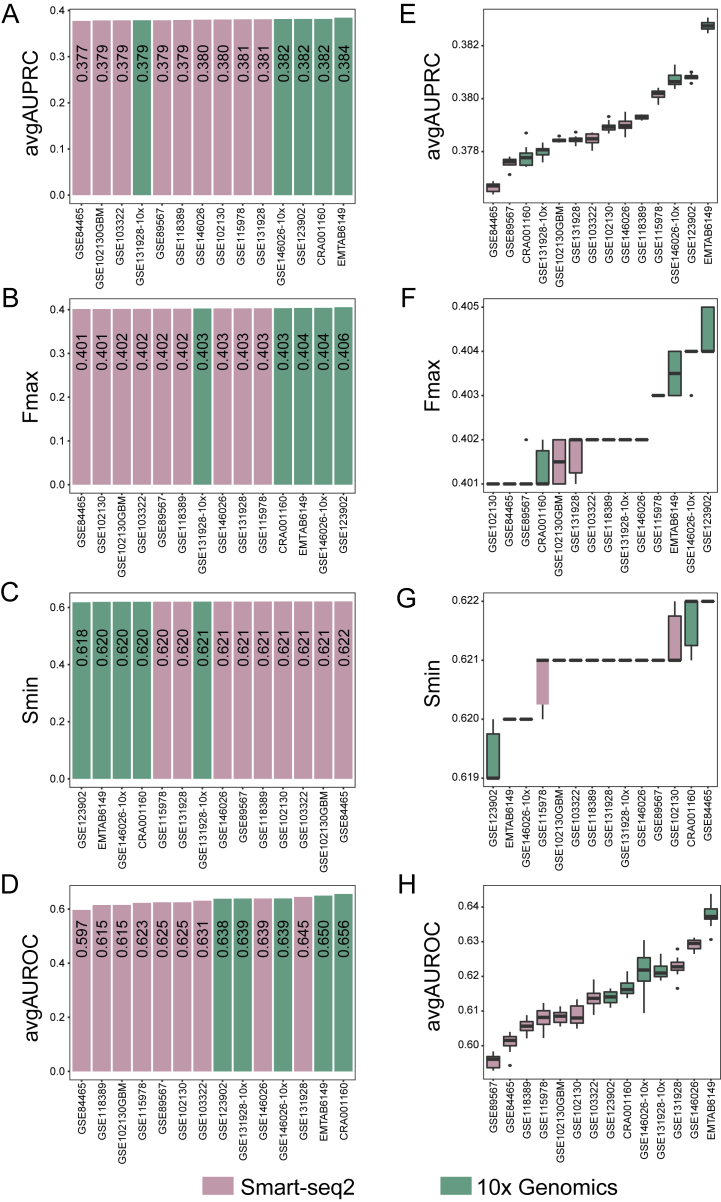
The impacts of the single-cell sequencing platform on the performance of gene function prediction. (A–D) The performance of scRNA-seq datasets from Smart-seq2 or 10x Genomics. (E–H) The performance of scRNA-seq datasets from Smart-seq2 or 10x Genomics after fixing the number of cells in single-cell expression profiles.

There are inherent differences between scRNA-seq datasets generated from different platforms, which may affect the calculation of co-expression and then the gene function prediction. Thus, we assessed the performance of gene function prediction of scRNA-seq profiles generated from Smart-seq2 and 10x Genomics, two of the mostly used scRNA-seq platforms. Here, only 2567 genes, which expressed in all the 14 scRNA-seq datasets, were used as the tested genes. Interestingly, the avgAUPRC values of scRNA-seq datasets from 10x Genomics were slightly higher than those of the scRNA-seq datasets from Smart-seq2 (Fig. 7A). The similar results were also observed for $F_{\max}$ (Fig. 7B) and avgAUROC (Fig. 7D). And the 10x datasets tend to have lower $S_{\min}$ scores than Smart-seq2 datasets (Fig. 7C). Considering the impact of cell number on the performance and the dataset with the least cells (GSE102130GBM [13], N=599), we constructed a new expression profile by randomly selecting 550 cells and calculated the prediction performance. To obtain the average performance, we repeated the calculation 10 times for each of the 14 scRNA-seq datasets. Again, 10X datasets still had a slightly higher performance than Smart-seq2 datasets (Fig. 7E–H). Taken together, scRNA-seq datasets generated from 10x Genomics have a better prediction performance than those from Smart-seq2. More importantly, the sequencing throughput of 10x Genomics is far higher than that of Smart-seq2, therefore, scRNA-seq datasets generated from 10x Genomics are more suitable for gene function prediction.

### 3.6 The impact of co-expression metrics, excessive zeros and negative correlations on the performance

Co-expression network is one of the most important foundations in gene function prediction. Thus, we next explored the impact of different methods for co-expression network construction on the performance of gene function prediction with single-cell data.

**Fig. 8:**
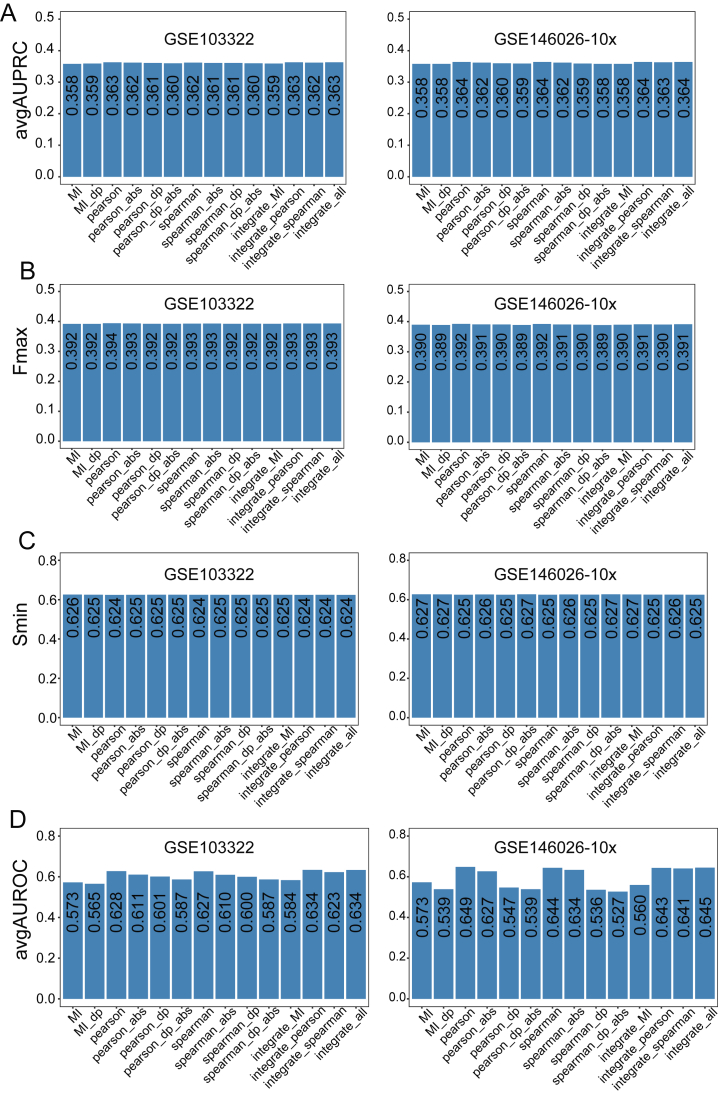
The impacts of the single-cell co-expression network on the performance of gene function prediction. (A–D) The overall performance of different co-expression networks.

Firstly, we explored the influence of three different co-expression metrics, including mutual information, Pearson correlation coefficient and Spearman rank correlation coefficient. Networks constructed based on Pearson and Spearman correlation coefficients had slightly higher avgAUPRC values, slightly higher $F_{\max}$ scores and slightly lower $S_{\min}$ scores than those constructed based on mutual information (Fig. 8A–C; Figs. S17–19). And networks constructed based on Pearson and Spearman correlation coefficients had higher avgAUROC values (Fig. 8D; Fig. S20). In summary, the simple correlation can capture the co-expression information, and this phenomenon has also been observed in bulk data [24].

Owing to the low mRNA content in individual cells, inefficient mRNA capture, and the stochasticity of mRNA expression, dropout events occurred frequently in single-cell sequencing, resulting in the zero-inflated single-cell expression profile, which may require special attention in traditional analysis such as correlation analysis. Thus, we next tested the impact of these excessive zeros on the performance by excluding them from the expression data when constructing co-expression network. From the view of genes, most of the co-expression networks with “0” showed slightly higher avgAUPRC values, slightly higher $F_{\max}$ scores and slightly lower $S_{\min}$ scores than networks without “0” (Fig. 8A–C; Figs. S17–19). And in term-centric evaluation, the avgAUROC values of co-expressed networks with "0″ were also higher than those of networks without "0" (Fig. 8D; Fig. S20). On the whole, considering “0” values in the single-cell expression profiles when constructing co-expression networks improved the performance of gene function prediction.

Next, we assessed the influence of the negative correlations by using the correlation coefficients themselves or their absolute values to rank the network edges. Both the gene-centric and term-centric evaluation showed that networks constructed by using the correlation coefficients themselves to rank the network edges had a better performance (Fig. 8; Figs. S17–20), which has also been reported in previous study [24].

Different co-expression networks described above may contain different types of information. So, whether integrating these individual networks can improve the prediction performance. The integrated single-cell networks had slightly higher avgAUPRC values, $F_{\max}$ scores and avgAUROC values, as well as slightly lower $S_{\min}$ scores than individual networks. Taken together, integrating individual networks can improve the performance of gene function prediction, but the improvement was limited (Fig. 8; Figs. S17–20)

## 4 Discussion

High functional heterogeneity of tumors poses a major challenge on cancer diagnosis and treatment. Accurate annotation of gene function in each patient, even in each cell type, is key to understanding the pathogenesis of cancers. Single-cell sequencing provides an unprecedented opportunity to decipher gene function at single-cell resolution. Thus, in this study, we constructed a function prediction framework based on co-expression network and neighbor-voting algorithm to reveal the unique value of scRNA-seq in understanding gene function.

A general performance evaluation in gene function prediction is far from straightforward. Several evaluation metrics were used in this study, because each provides useful insights and complements the others. In the GO annotations, the positive terms for a gene are far less than the negative ones. The PR curve, which only calculates the positive instances rather than the negative ones, is the most suitable for such highly imbalanced data. And meanwhile, it has high interpretability: if, for a given threshold, the precision and recall are 0.6 and 0.7, respectively, this indicates that on average 60 % of the predicted terms will be correct and that about 70 % of the true terms will be recalled. PR curves treat all the terms equally, without considering the differences between terms, such as the information content. The RU-MI curve considers the information content of each GO terms besides counting the number of true positives, false positives, etc. It lowers the weights of the low-information terms, as the ability to predict such low-information content terms is not as desirable and useful as the ability to predict terms with high-information content. However, the RU-MI curve is somehow less interpretable than the PR curve. Therefore, we used both the PR curve and RU-MI curve in gene-centric evaluation to achieve a more comprehensive view of the performance. The term-centric evaluation is an example of binary classification, in which a given term is assigned (or not) to an input gene. The ROC curve is the most widely used performance metric for the evaluation of binary classification models because of several well-studied characteristics, such as intuitive visual interpretation, convenience in comparing multiple models, and the single-value quantity, AUROC [36,37]. Moreover, it considers both positive and negative instances and is suitable for overall performance evaluation [36]. Thus, the ROC curve was used in the term-centric evaluation to assess the performance.

By comparing 14 scRNA-seq datasets with bulk RNA-seq datasets of the same cancer types, we found that, on the whole, the performance of single-cell and bulk was comparable. In addition, in our study, $F_{\max}$ values ranged from 0.389 to 0.399; $S_{\min}$ values ranged between 0.622 and 0.627; and the average AUROC ranged from 0.612 to 0.678 (Fig. S2). In previous studies, $F_{\max}$ and $S_{\min}$ values ranged from 0.25 to 0.40 and from 0.55 to 0.66, respectively; and the average AUROC around 0.70 [[27], [28], [29]]. Thus, although the performance was not as good as we expected, it was comparable to that of previous studies. Furthermore, single-cell expression profiles recalled more experimentally validated functions. This may be because of the high resolution and throughput of single-cell sequencing. In studies based on scRNA-seq, it was not difficult to find that many genes only expressed in some cells, suggesting that these genes may play a role only in these cells. For a gene, scRNA-seq can detect its expression in each cell, while bulk RNA-seq detects its average expression in all cells, which conceals the differences in its expression between different cells. In this study, we found that the number of cells had a great impact on the prediction performance. Generally, the number of cells in a single-cell dataset was much more than the number of samples in a bulk dataset. Therefore, it may be easier to reveal the change trend of expression between genes in single-cell sequencing than in bulk sequencing, so as to recall more experimentally validated functions.

Importantly, the single-cell expression profile of immune cells recalled immune-related functions, which were not discovered by tumor cells. And the single-cell expression profiles of individual samples discovered sample-specific functions. These results indicated the contribution of single-cell sequencing to the understanding of gene function and functional heterogeneity in cancers. Moreover, the performance of the scRNA-seq data from several samples was comparable to that of the bulk RNA-seq data from hundreds of samples, suggesting that gene function prediction based on scRNA-seq can save samples and overcome a series of problems caused by sampling difficulties.

In order to find out the attention points for using scRNA-seq to predict gene function, we systematically evaluated the factors that may affect the performance and observed several meaningful phenomena. (1) We found the prediction performance increased with the number of cells, and 500 or more cells should be considered for gene function prediction with scRNA-seq. (2) The scRNA-seq dataset generated from 10x Genomics had a better performance than that generated from Smart-seq2. (3) Considering “0” values in the single-cell expression profiles when constructing co-expression networks improved the performance, suggesting that these zeros in the expression profile may have some biological significance. (4) The simple correlation can capture the co-expression information. (5) Genes positively correlated with each other were more likely to participate in the same biological process than the negative ones. And the latter two results have been reported in previous studies with bulk RNA-seq [24].

In summary, combining gene-centric and term-centric evaluation, we compared the prediction performance of bulk data and scRNA-seq data from multiple perspectives. The results revealed the irreplaceable role of single-cell sequencing in decoding the functional heterogeneity of genes in cancers, which was of great significance in determining the pathogenesis of cancers more accurately, and so as to develop precision therapies. Simultaneously, we explored the factors that may affect the prediction performance, so as to give some suggestions to the follow-up studies.

However, there were several limitations in this study.

It has been reported that protein and mRNA levels typically showed reasonable correlation [38], and the transcriptome analysis can be used as a tool to predict the protein level [39]. However, the protein is the final product that performs functions. Thus, combining scRNA-seq data with different omics levels, such as scATAC-seq and CITE-seq data, may be able to improve the prediction performance effectively.

Accumulating evidence showed genes with similar expression patterns were likely to be involved in the same biological processes [[1], [2], [3], [4], [5]]. In Gene Ontology, Biological Process refers to a biological objective to which the gene or gene product contributes. A Biological Process is accomplished by the coordinated and ordered expression of the involved genes. Molecular Function is defined as the biochemical activity of a gene product, such as binding or catalysis, which may be more suitable for sequence-based function prediction methods. Cellular Component refers to the place in the cell where a gene product is active, and it is currently rarely used in gene function prediction based on co-expression [40]. Therefore, in this study, we only predicted the biological processes in which genes were involved. In future studies, Molecular Function and Cellular Component should be taken into account when combining multi-level omics data, thereby providing more comprehensive information on gene function.

In this study, we only used the scRNA-seq datasets from 10X Genomics and Smart-seq2 platforms, although the two platforms are the most widely used at present, we should also include scRNA-seq datasets generated by other platforms such as CEL-seq2.

In addition, we discussed three aspects that may affect the performance of gene function prediction based on scRNA-seq data, including the treatment of zero values in expression profiles, the number of cells, and the sequencing protocol. However, since only the processed data of most scRNA-seq data used currently are provided, it is impossible to further explore the impact of factors such as sequencing depth and read alignment rate on predicting gene function using scRNA-seq data.

Finally, it should be noted that there are currently some deconvolution algorithms, such as CIBERSORTx [41], which can infer cell type-specific expression profiles for each sample. Theoretically, such profiles can also be used to study cell type-specific gene functions. In this study, however, we did not evaluate the prediction efficiency of gene functions based on these cell type-specific expression profiles. This is undoubtedly an area deserving further exploration. And in the follow-up research, we will take it into key consideration, hoping to provide more valuable insights in this field.

## CRediT authorship contribution statement

**Huating Yuan:** Writing – original draft, Visualization, Validation, Resources, Methodology, Investigation, Funding acquisition, Formal analysis, Data curation, Conceptualization. **Xin Liang:** Visualization, Validation, Data curation. **Xinxin Zhang:** Writing – review & editing, Supervision, Project administration, Funding acquisition. **Yu Cao:** Writing – review & editing, Supervision, Project administration, Conceptualization.

## Availability of data

Publicly available datasets were analyzed in this study. These datasets were presented in the main text and in the Supplementary Material.

## Availability of code

The core code has been submitted to the Github website, and the link is https://github.com/HuatingYuan/PredictGeneFunction.

## Funding

This work was supported by the 10.13039/501100001809National Natural Science Foundation of China (32360158); the High-level Talents Startup Fund of 10.13039/501100010265Guizhou Medical University (J[2021]040); the Young Scholars Program of Education Department of Guizhou Province (KY[2022]243); the Science and Technology Fund of 10.13039/100017957Guizhou Provincial Health Commission (gzwkj2022-269); the Basic Research Program of Guizhou Science and Technology Department (ZK[2024]117); the 10.13039/501100002858China Postdoctoral Science Foundation (2020M681118); the Heilongjiang Postdoctoral Foundation (LBH-Z20166); and the Fundamental Research Funds for the Provincial Universities of Heilongjiang (2020-KYYWF-1426).

## Declaration of competing interest

The authors declare that they have no known competing financial interests or personal relationships that could have appeared to influence the work reported in this paper.
