# Supplementary material for: Single-cell transcriptomes reveal cell-type-specific and sample-specific gene function in human cancer
Source: Heliyon. 2025 Jan 23;11(3):e42218. doi: 10.1016/j.heliyon.2025.e42218 (PMC11830296; doi:10.1016/j.heliyon.2025.e42218)
Supplement: Multimedia component 1 [file mmc1.docx]

***Supplementary Material***

# Supplementary Figures and Tables

## 1.1 Supplementary Figures


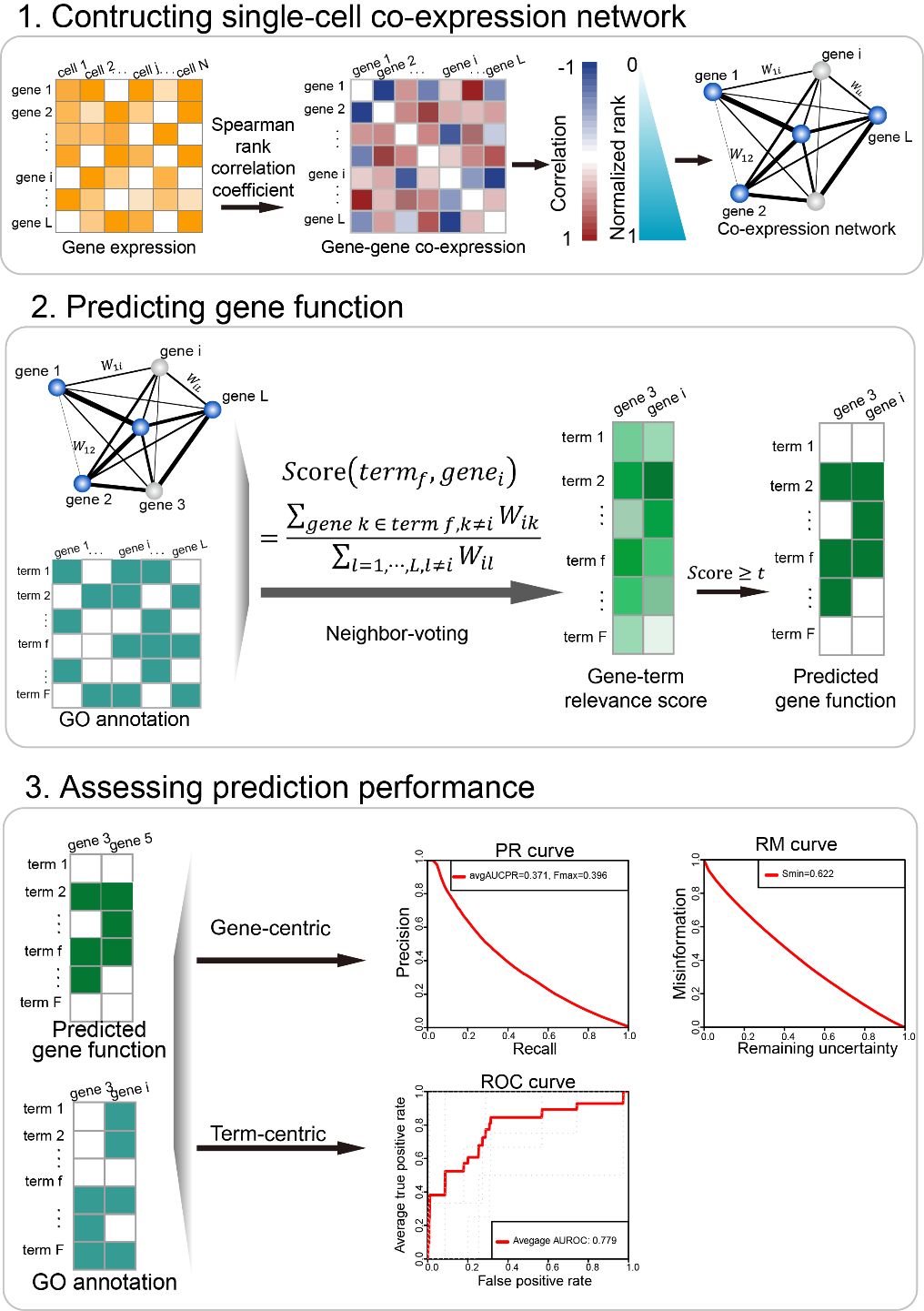


**Figure S1.** The framework of gene function prediction. The weighted co-expression network was constructed by calculating the Spearman rank correlation coefficients based on the expression profile. Then, the correlation coefficients were ranked and normalized to between 0 and 1, and were defined as the weights of edges. The functional relevance scores between genes and GO terms were measured using the neighbor-voting method. And a gene was predicted to relate to a term when the relevance score was greater than or equal to a given threshold $t$. Two modes of evaluations were used here. In gene-centric evaluation, the PR curve and RU-MI curve were used to assess the performance. The ROC curve was used in term-centric evaluation to assess the performance.


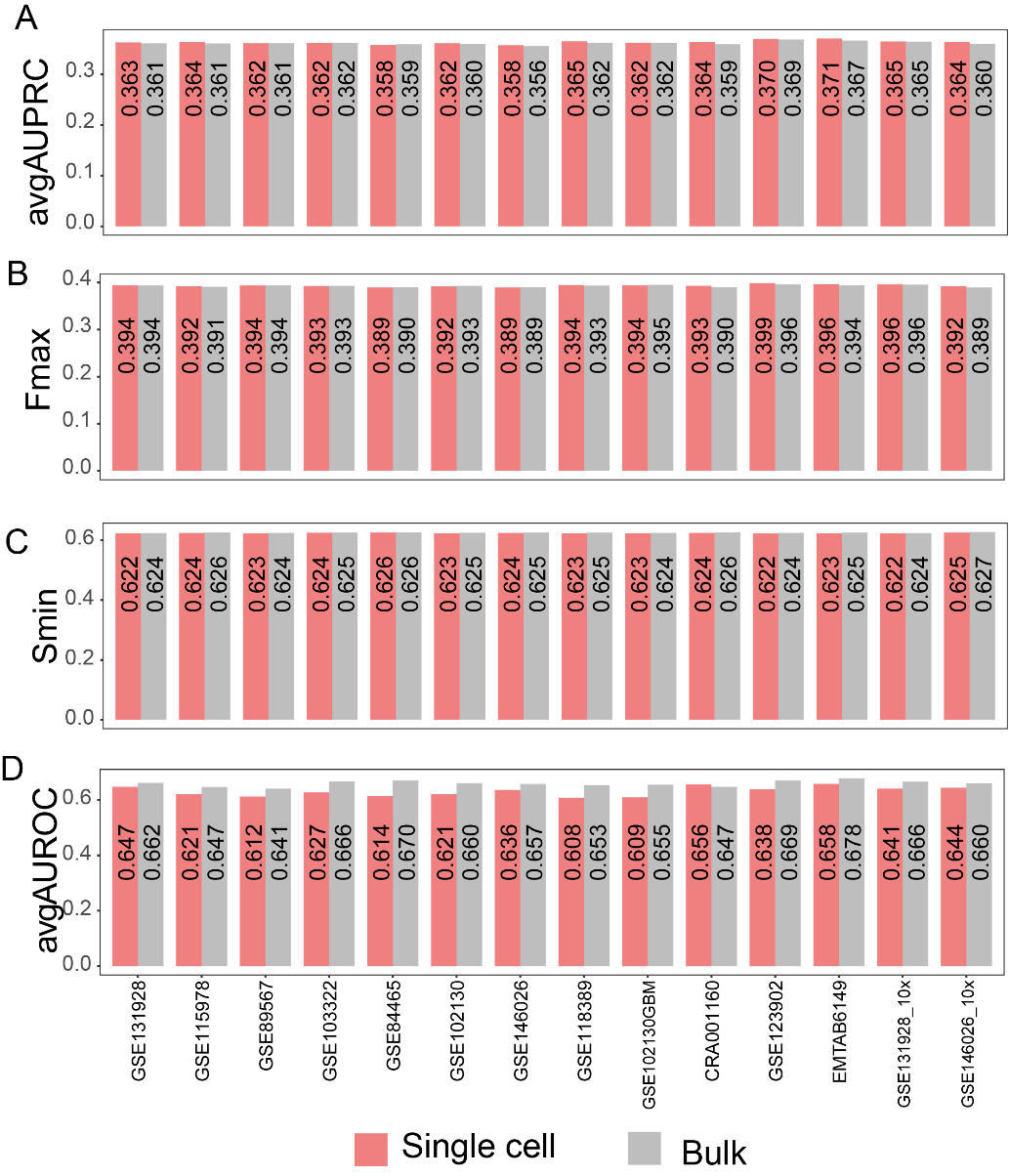


**Figure S2.** The overall performance of scRNA-seq datasets and bulk RNA-seq datasets.


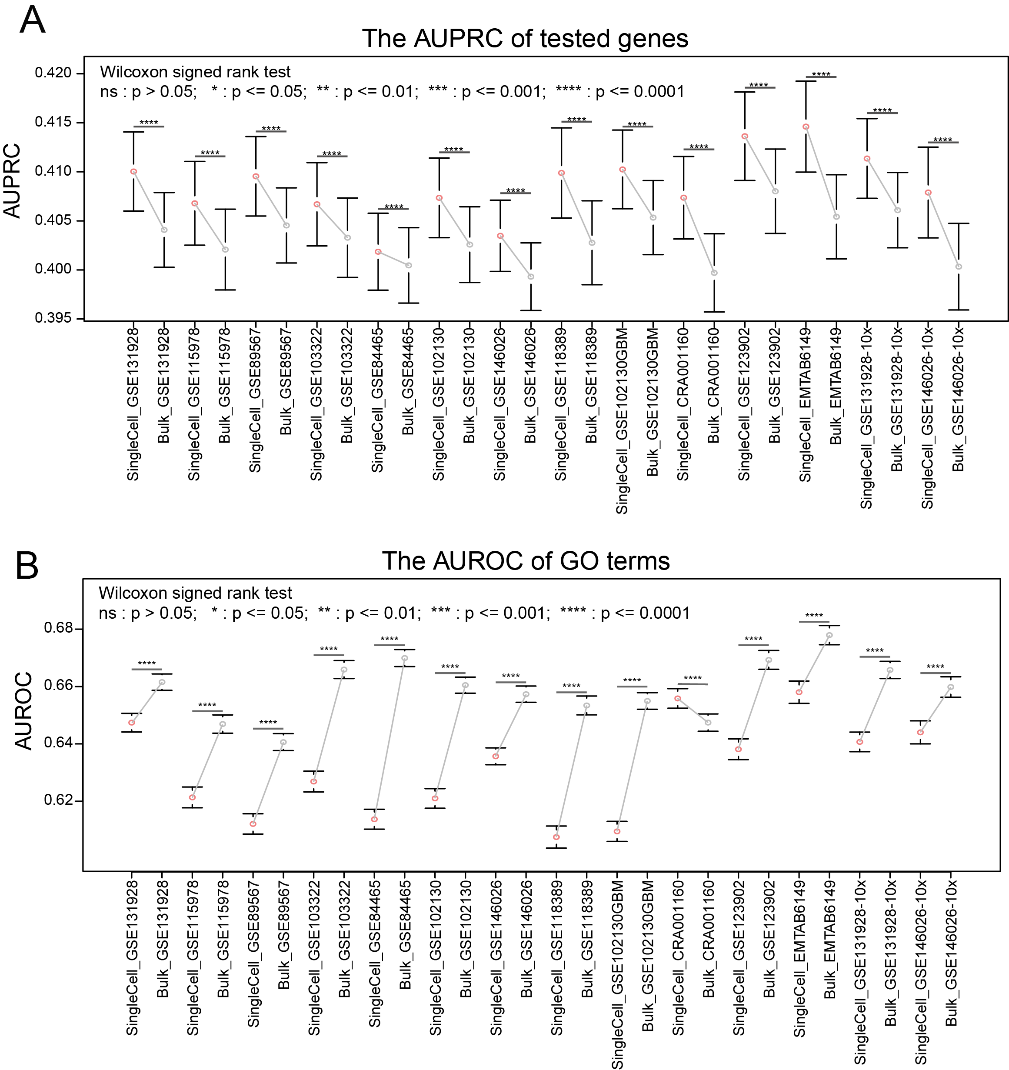


**Figure S3.** (A) The difference in AUPRC values of tested genes between scRNA-seq and bulk. (B) The difference in AUROC values of terms between scRNA-seq and bulk.


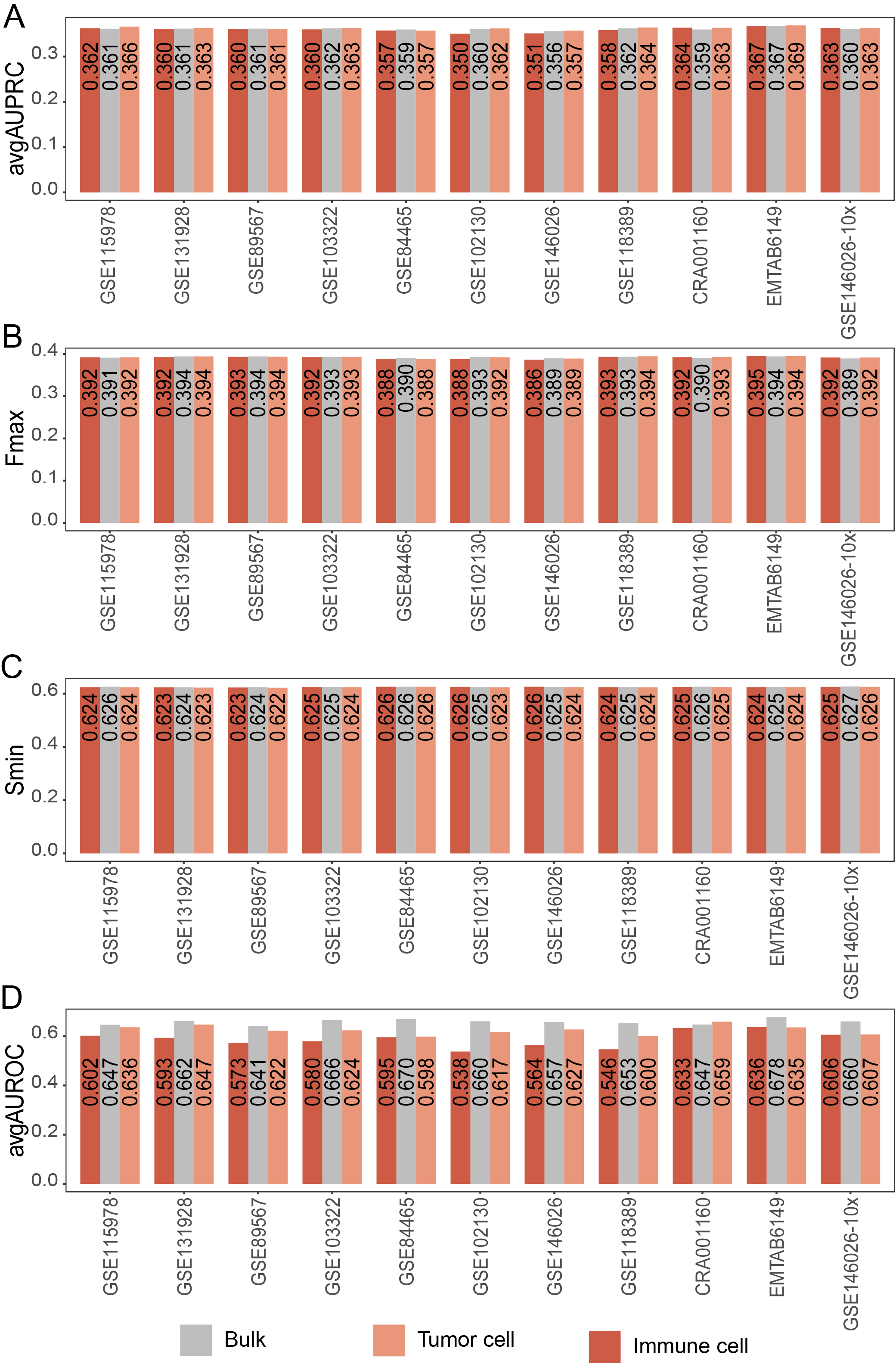


**Figure S4.** The overall performance of scRNA-seq data of immune or tumor cells and bulk RNA-seq data.


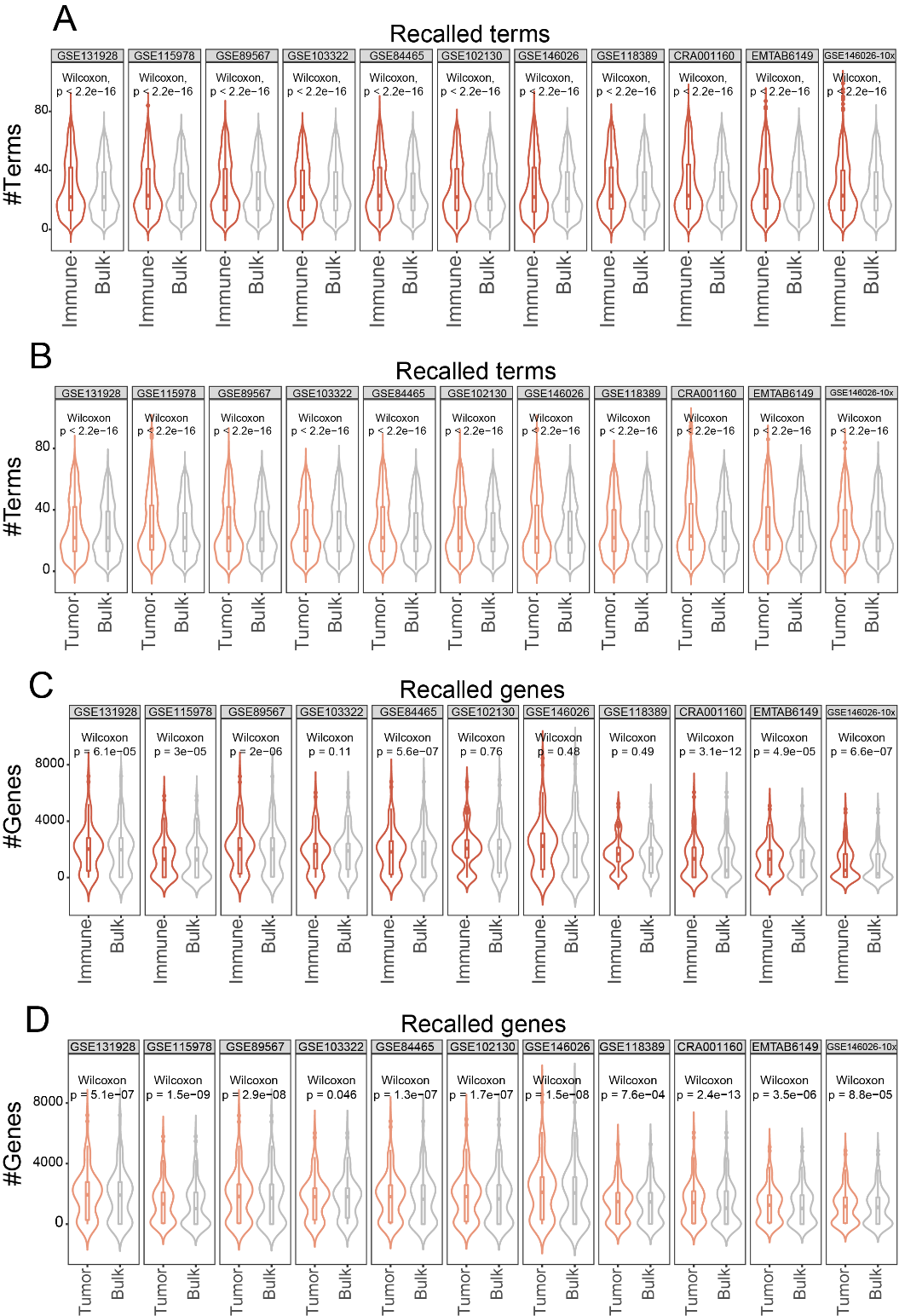


**Figure S5.** (A) The number of recalled terms discovered in immune cells or bulk. (B) The number of recalled terms discovered in tumor cells or bulk. (C) The number of recalled genes discovered in immune cells or bulk. (D) The number of recalled genes discovered in tumor cells or bulk.


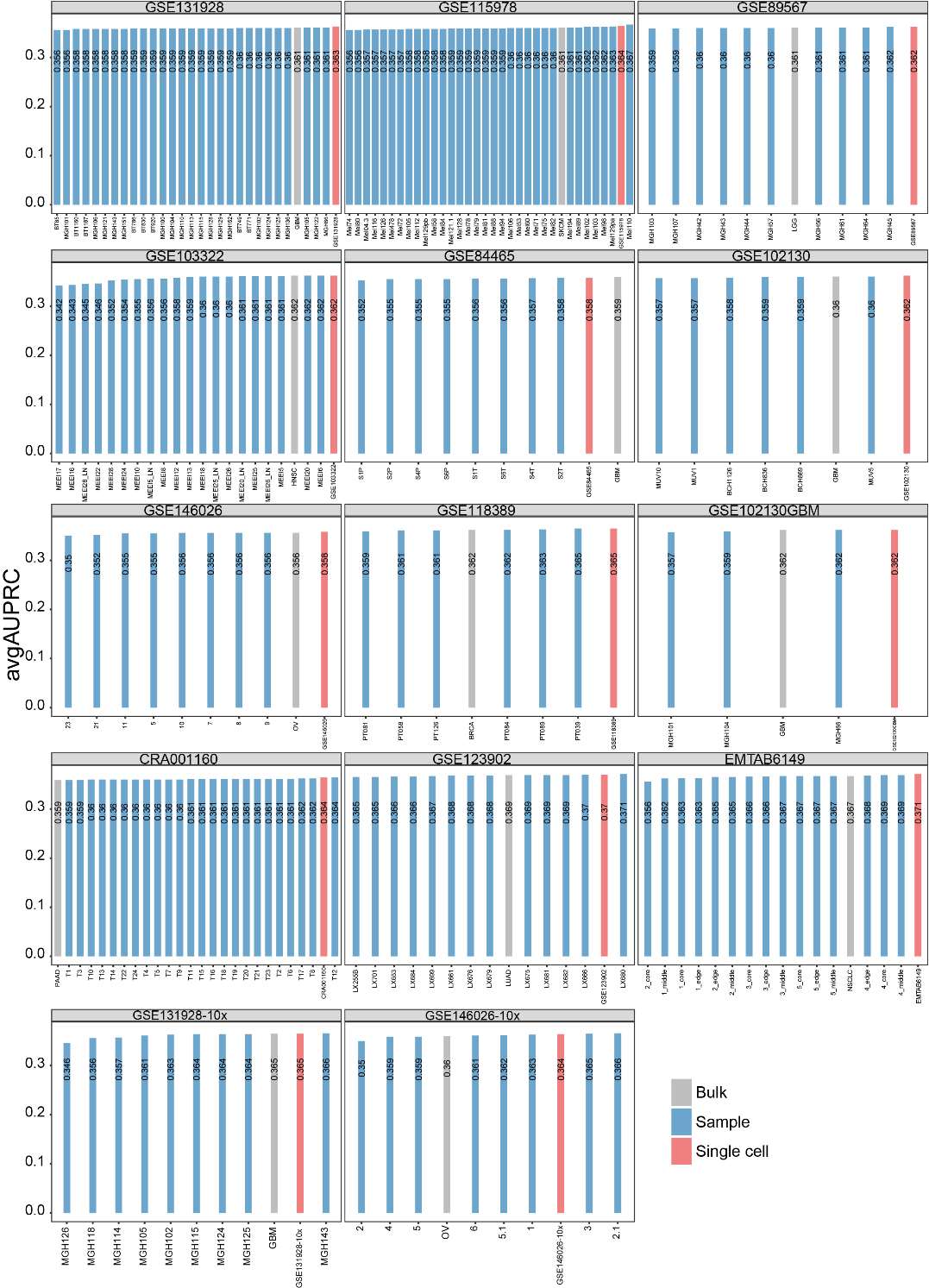


**Figure S6.** The avgAUPRC values of individual samples and bulk.


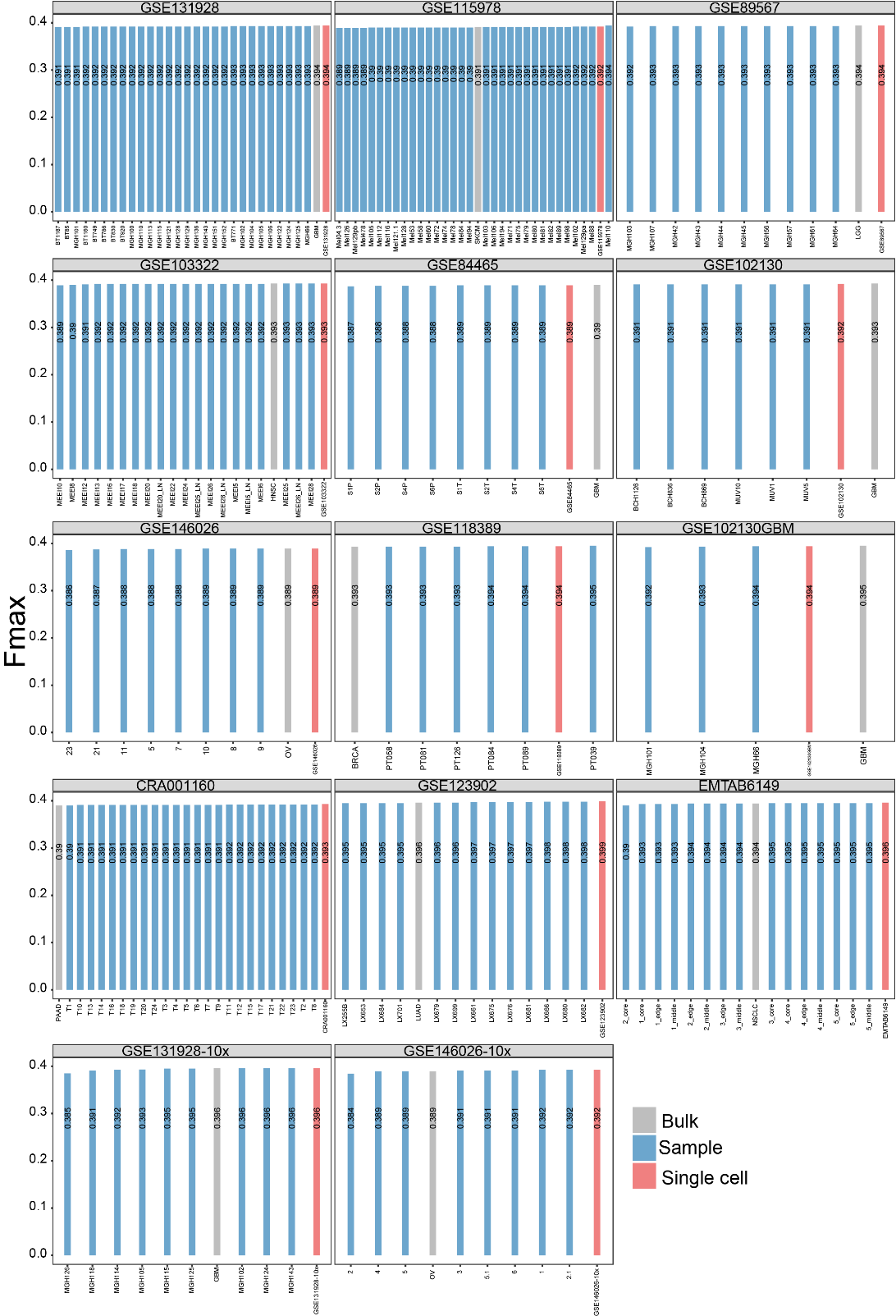


**Figure S7.** The $F_{max}$ scores of individual samples and bulk.


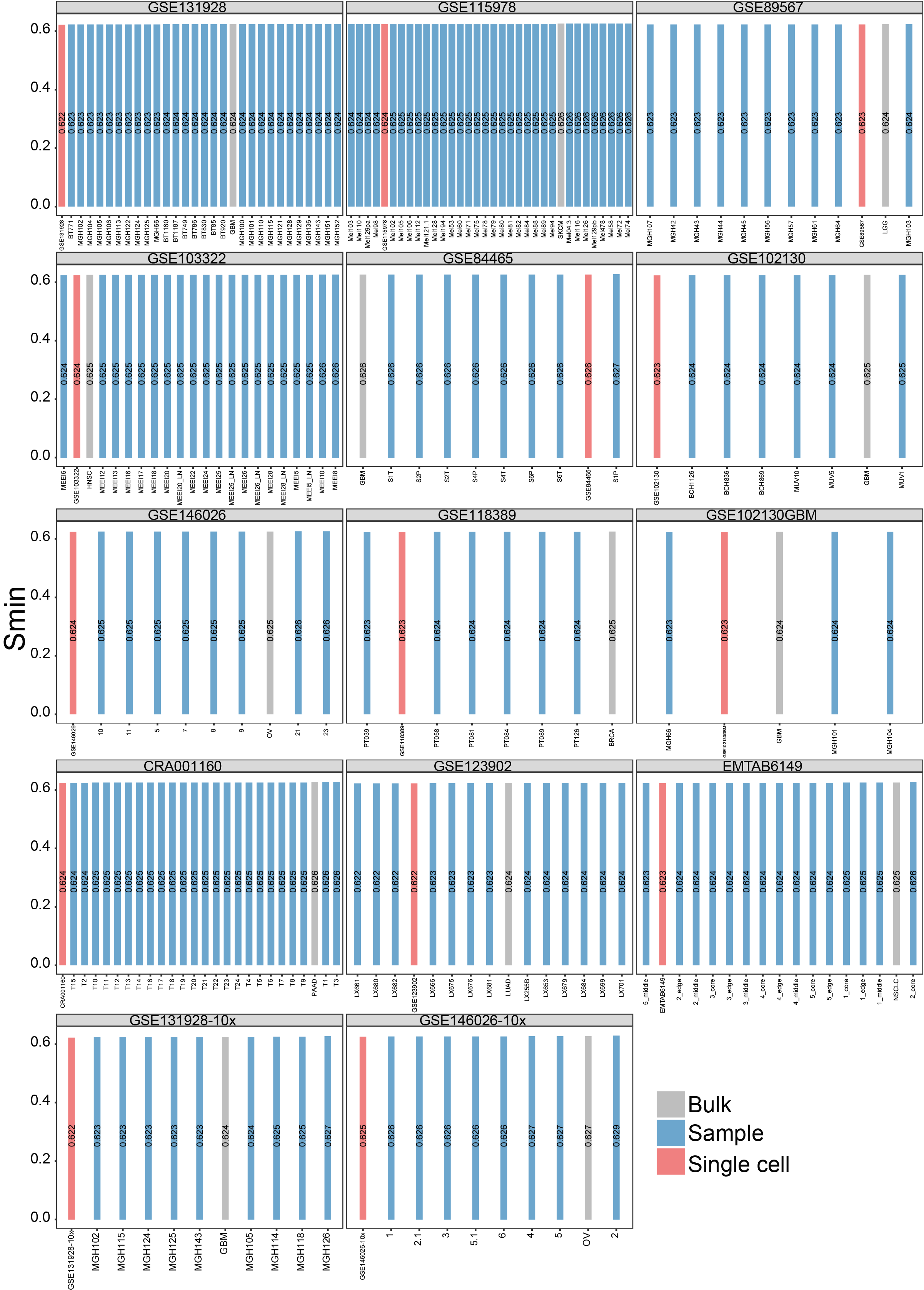


**Figure S8.** The $S_{min}$ scores of individual samples and bulk.


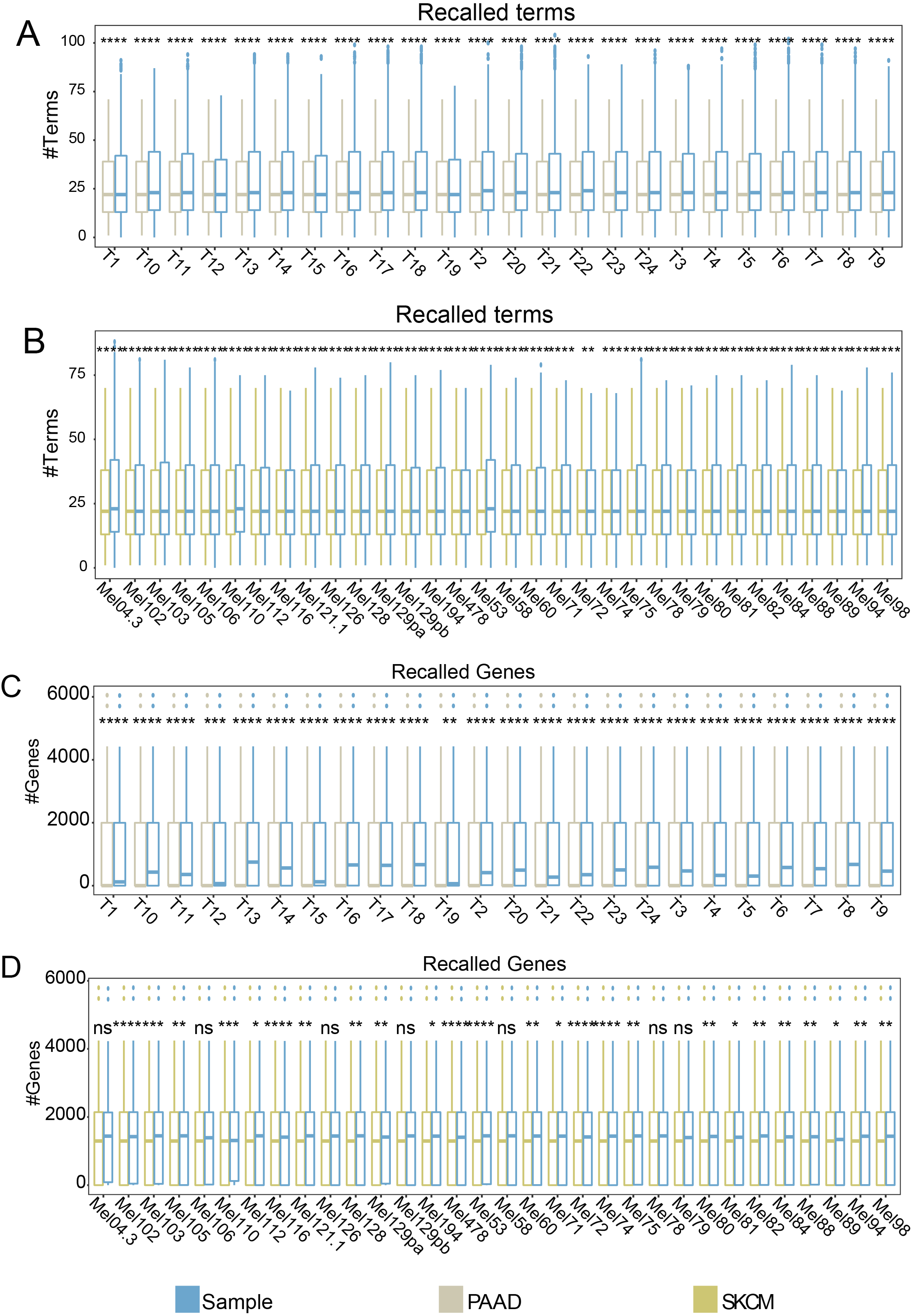


**Figure S9.** (A) The number of recalled terms discovered in PAAD or in individual samples from CRA001160. (B) The number of recalled terms discovered in SKCM or in individual samples from GSE115978. (C) The number of recalled genes discovered in PAAD or in individual samples from CRA001160. (D) The number of recalled genes discovered in SKCM or in individual samples from GSE115978.


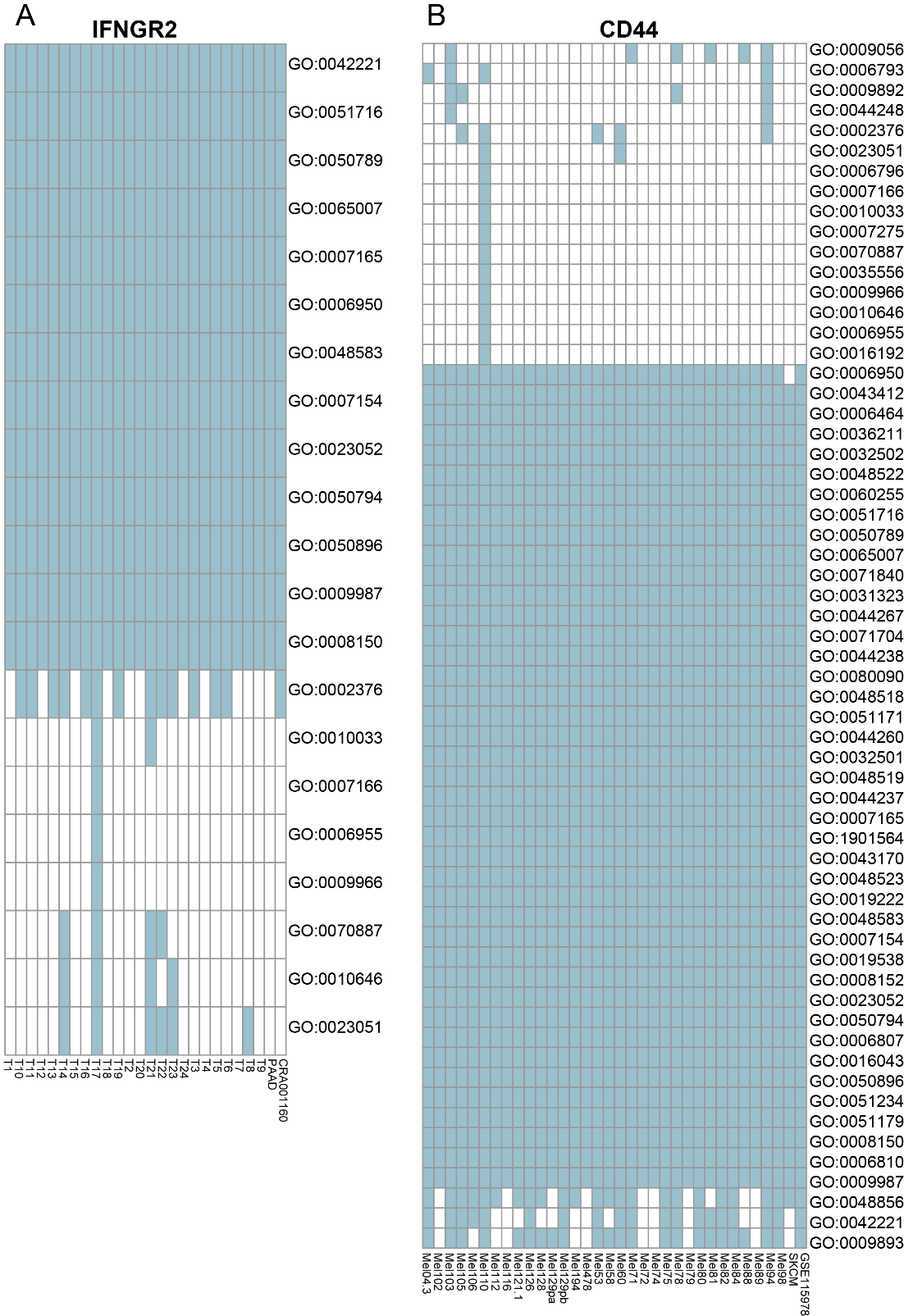


**Figure S10.** The experimentally validated terms recalled in individual samples or bulk. (A) The experimentally validated terms of IFNGR2 recalled in PAAD, CRA001160 or individual samples of CRA001160. (B) The experimentally validated terms of CD44 discovered in SKCM, GSE115978 or individual samples of GSE115978.


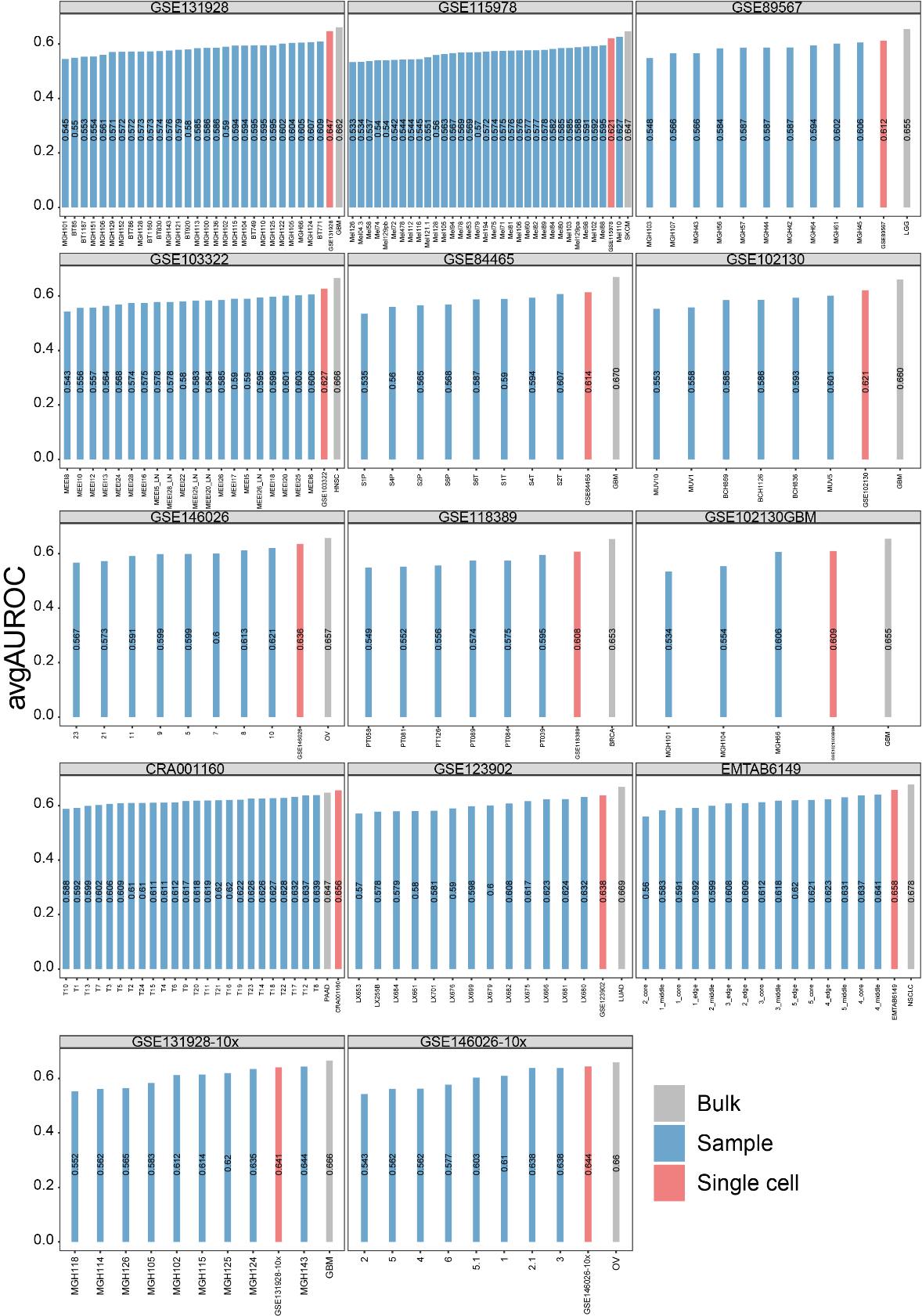


**Figure S11.** The avgAUROC values of individual samples and bulk.


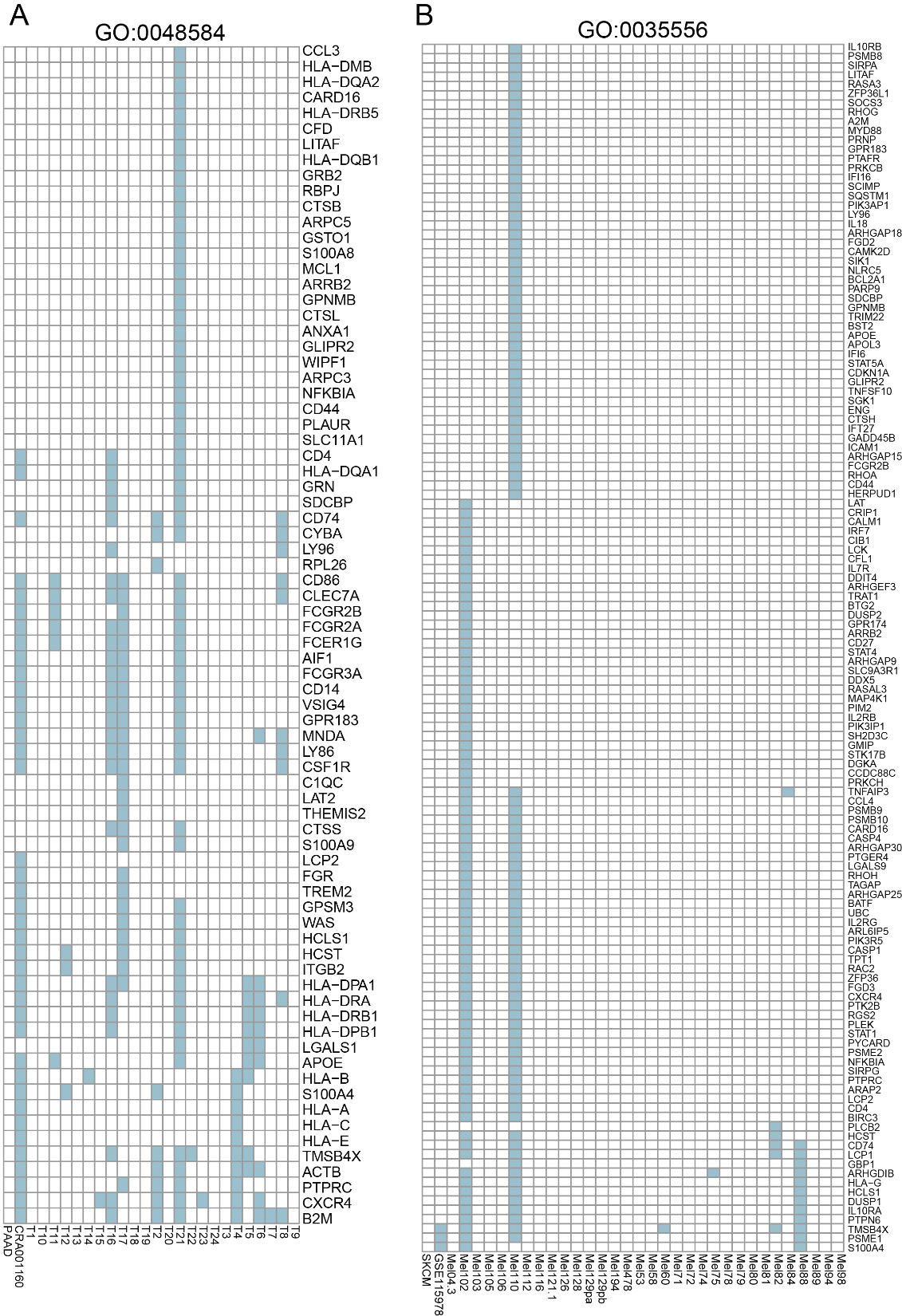


**Figure S12.** The experimentally validated genes discovered in individual samples or bulk. (A) The experimentally validated genes of “GO:0048584” recalled in PAAD, CRA001160 or individual samples of CRA001160. (B) The experimentally validated genes of “GO:0035556” discovered in SKCM, GSE115978 or individual samples from GSE115978.


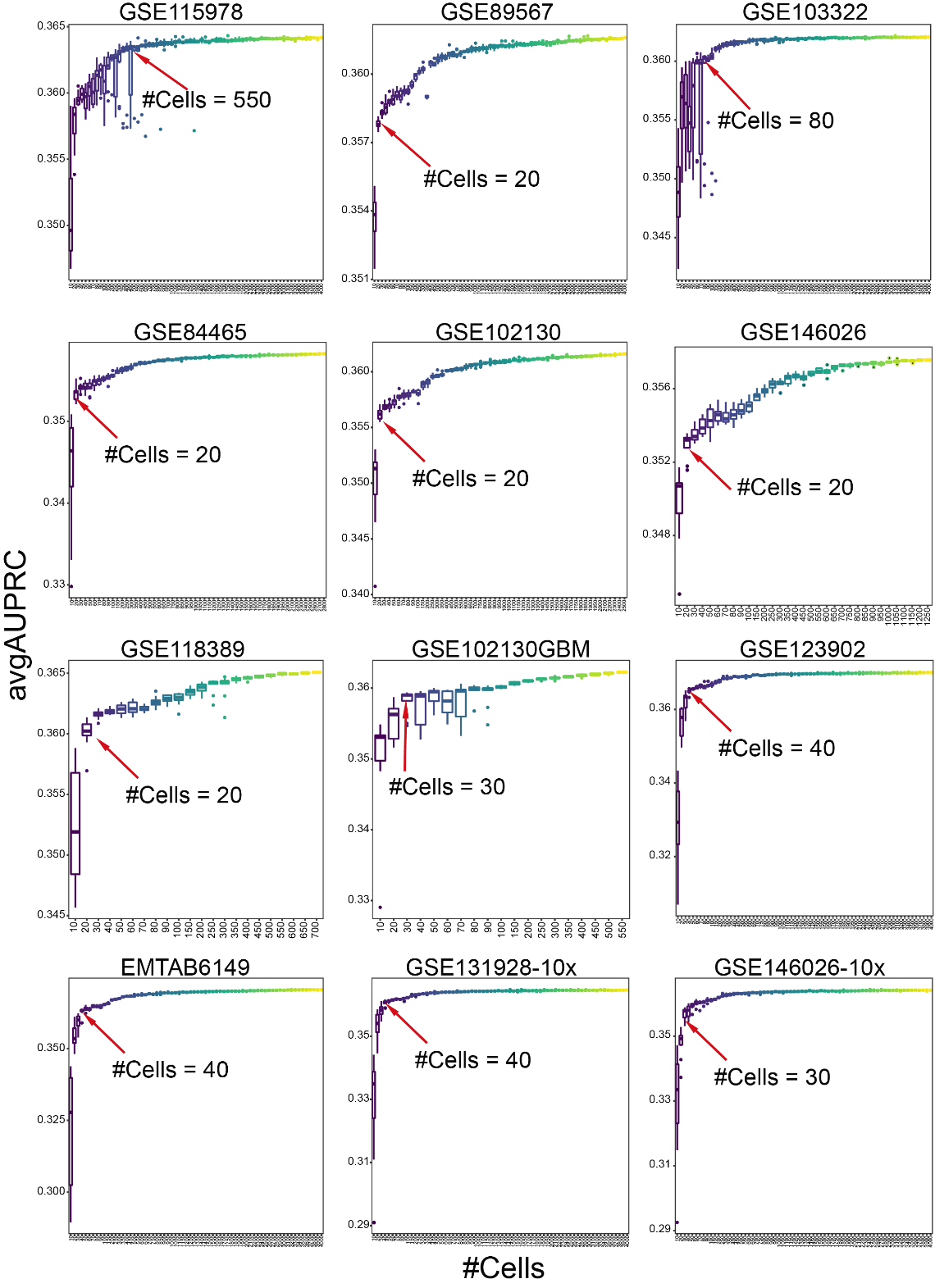


**Figure S13.** The impact of cell number on the avgAUPRC value of gene function prediction.


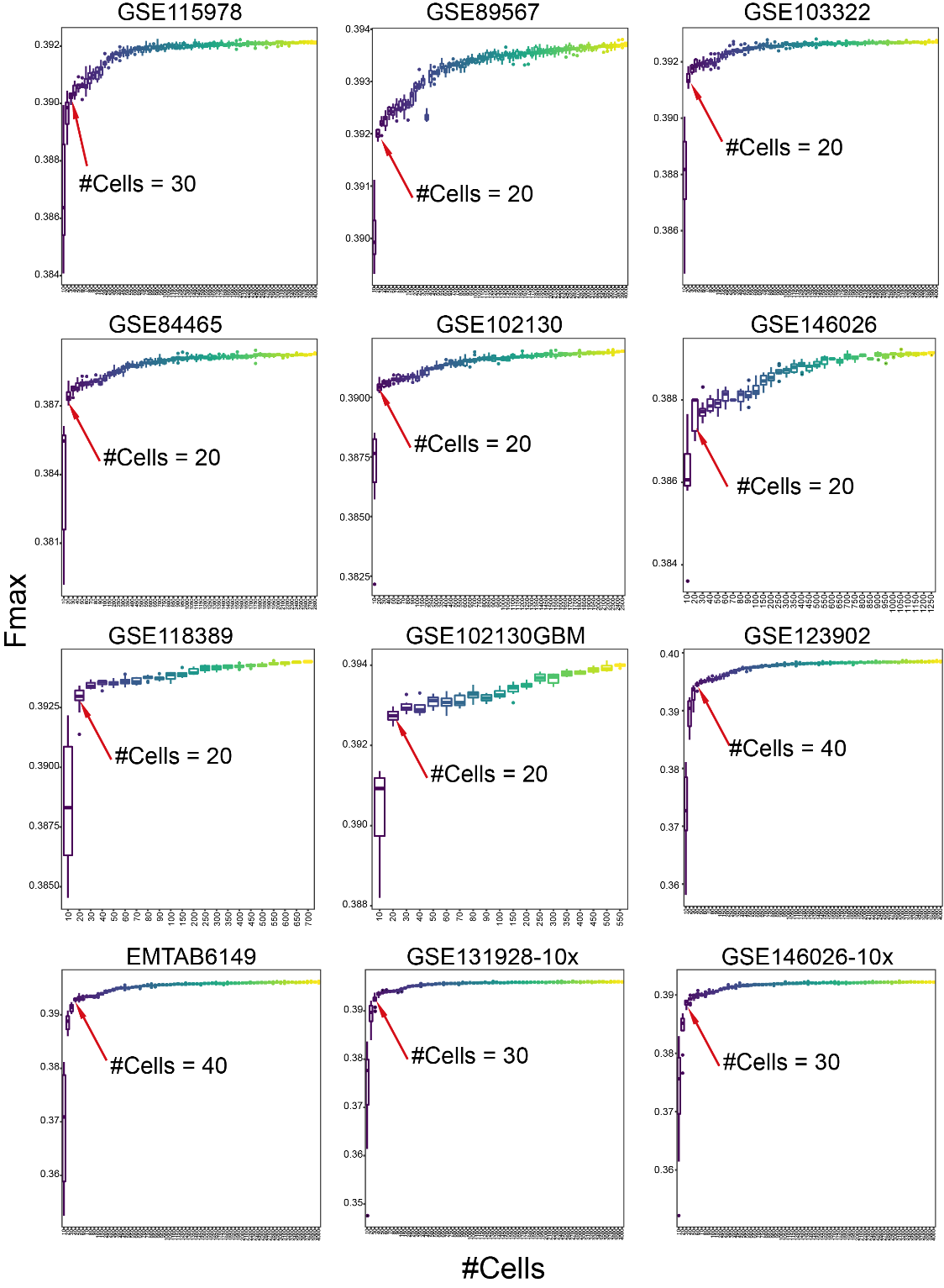


**Figure S14.** The impact of cell number on the $F_{max}$ score of gene function prediction.


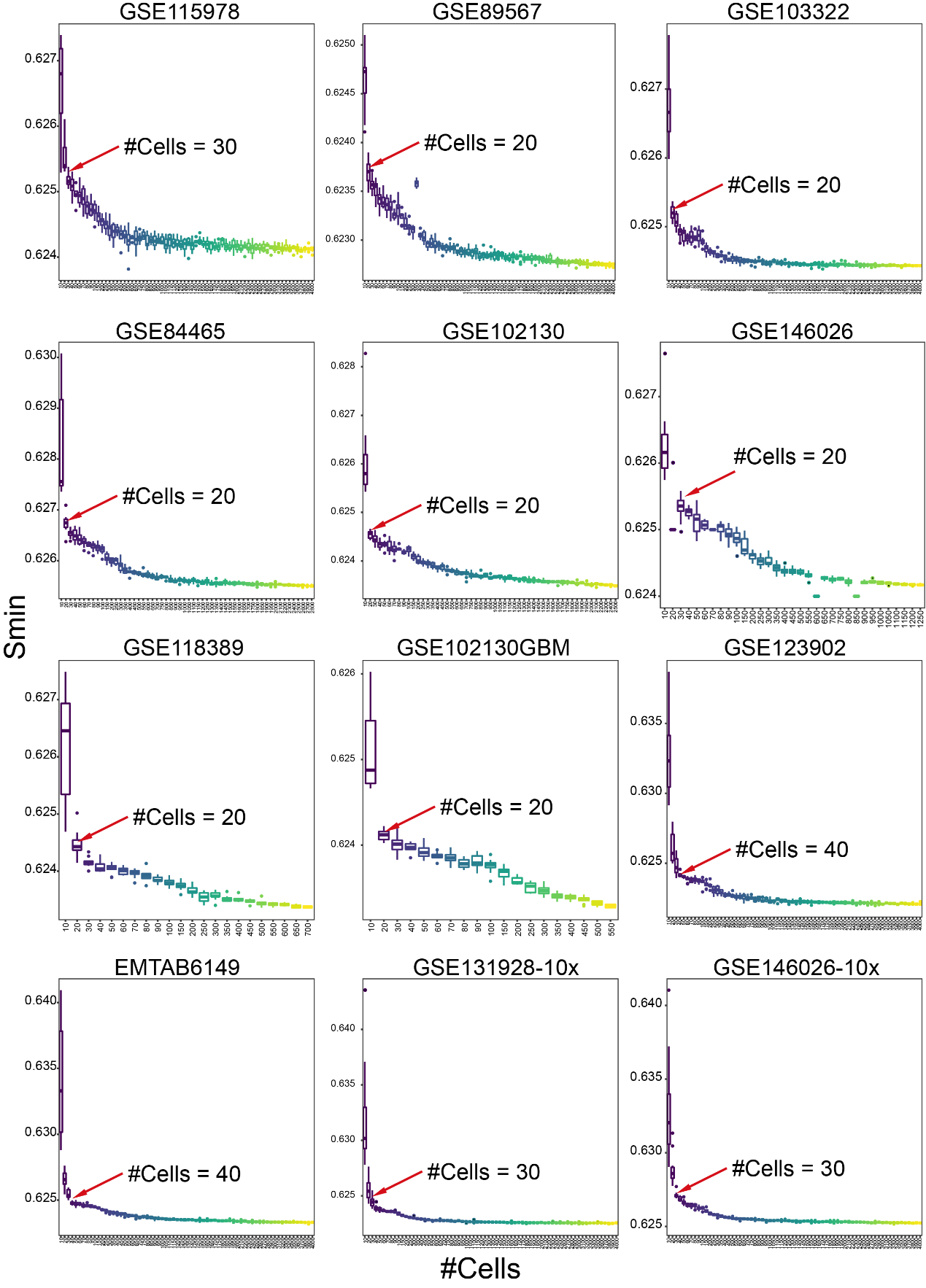


**Figure S15.** The impact of cell number on the $S_{min}$ score of gene function prediction.


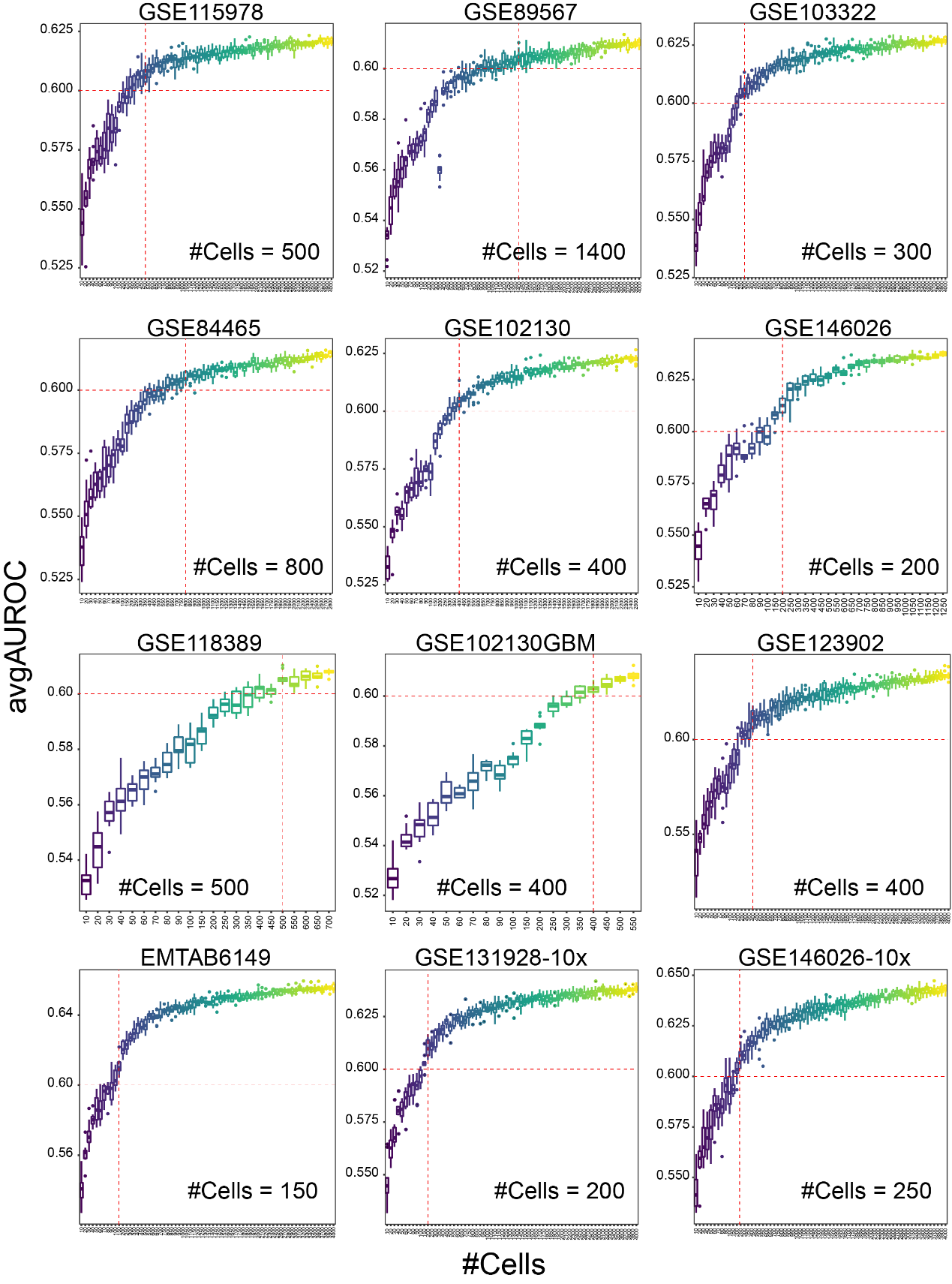


**Figure S16.** The impact of cell number on the avgAUROC value of gene function prediction.


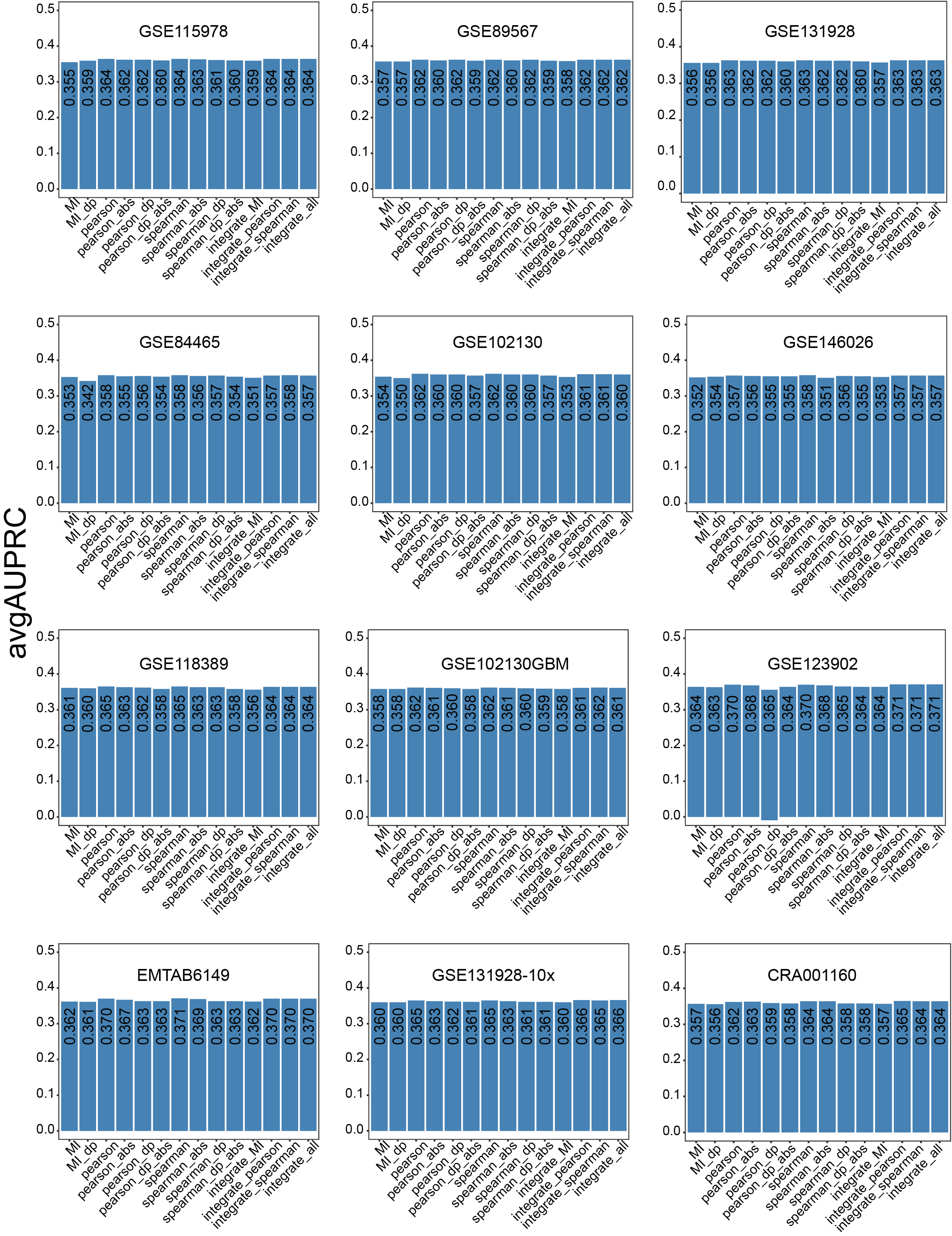


**Figure S17.** The avgAUPRC values of gene function prediction based on different single-cell co-expression networks.


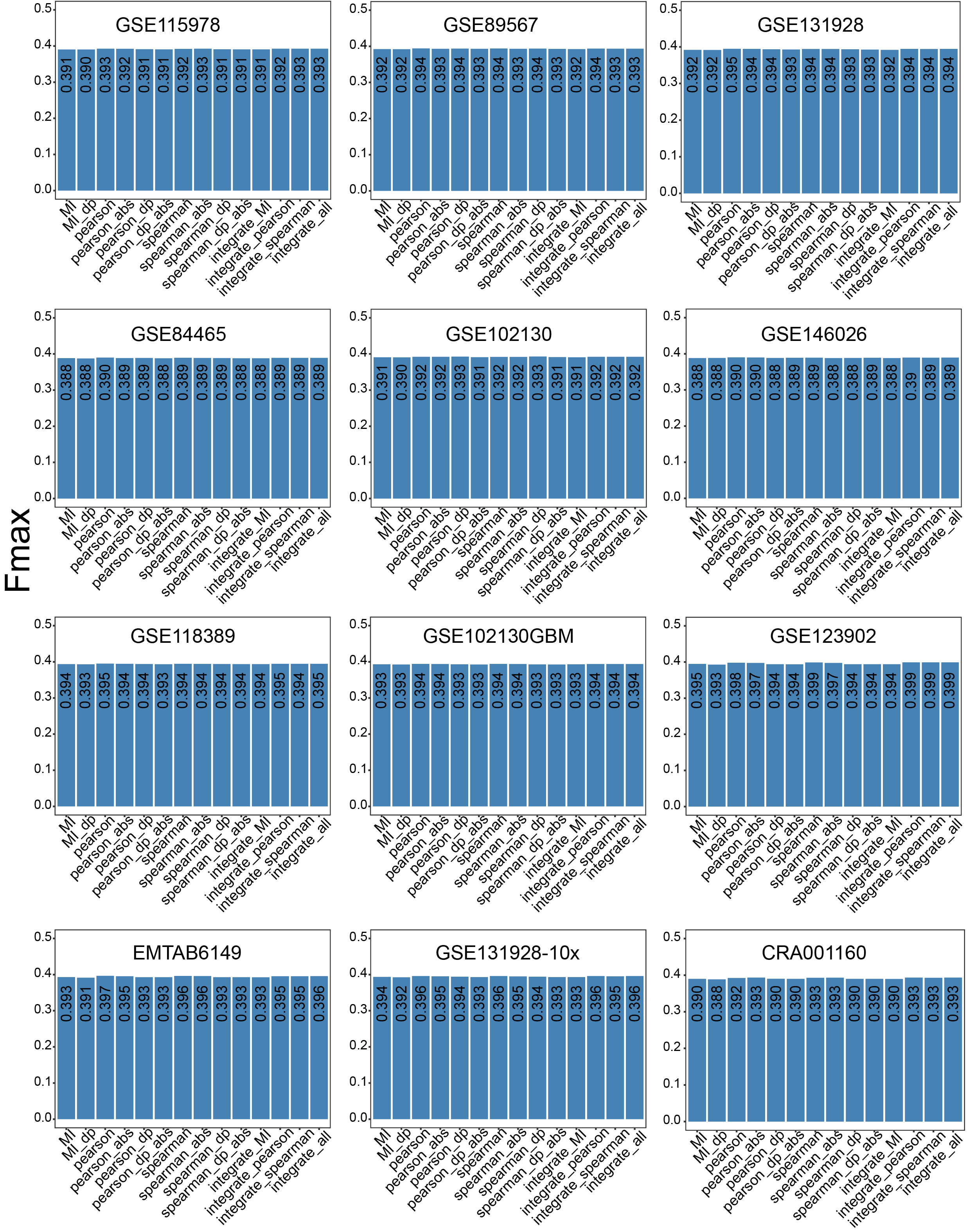


**Figure S18.** The $F_{max}$ scores of gene function prediction based on different single-cell co-expression networks.


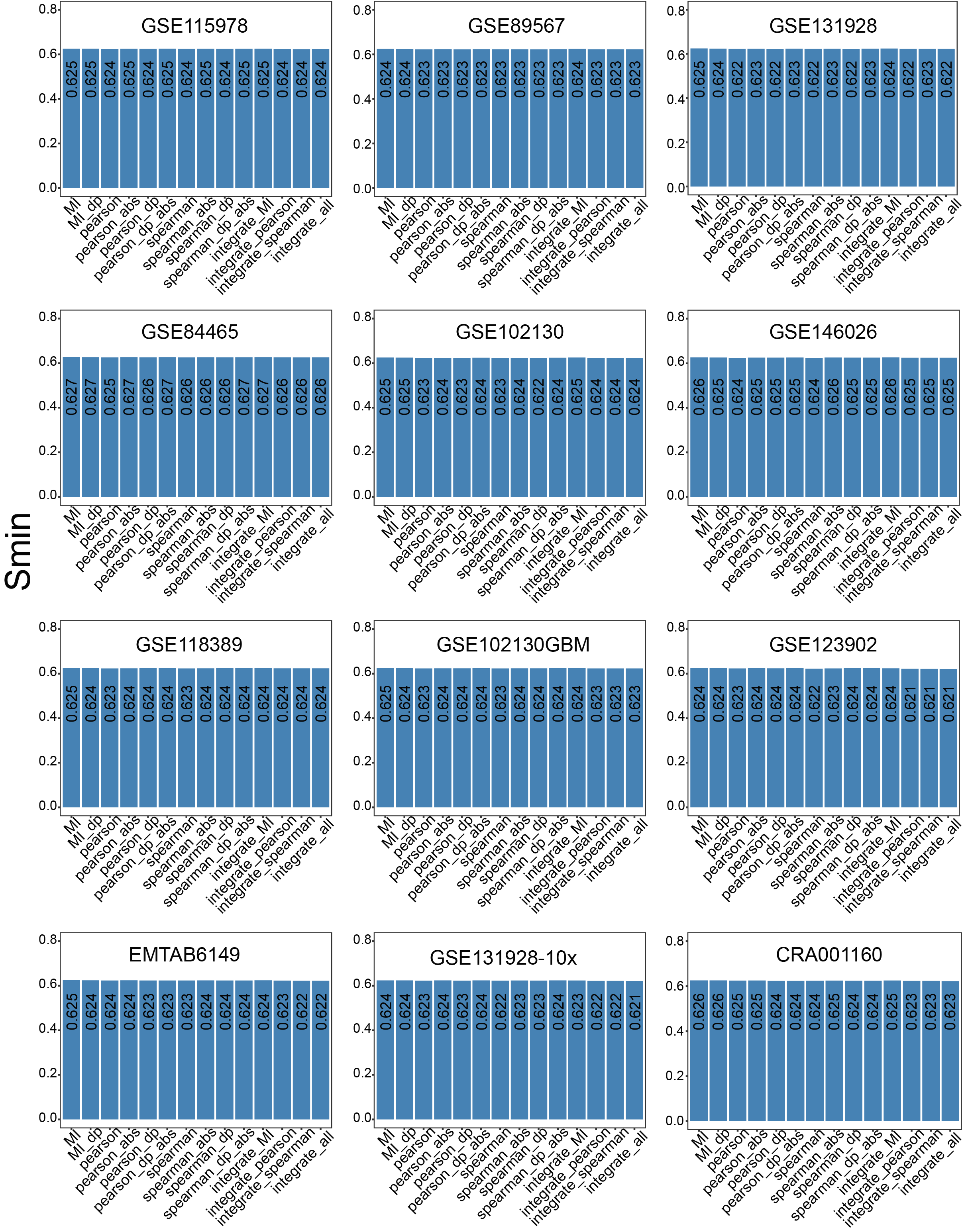


**Figure S19.** The $S_{min}$scores of gene function prediction based on different single-cell co-expression networks.


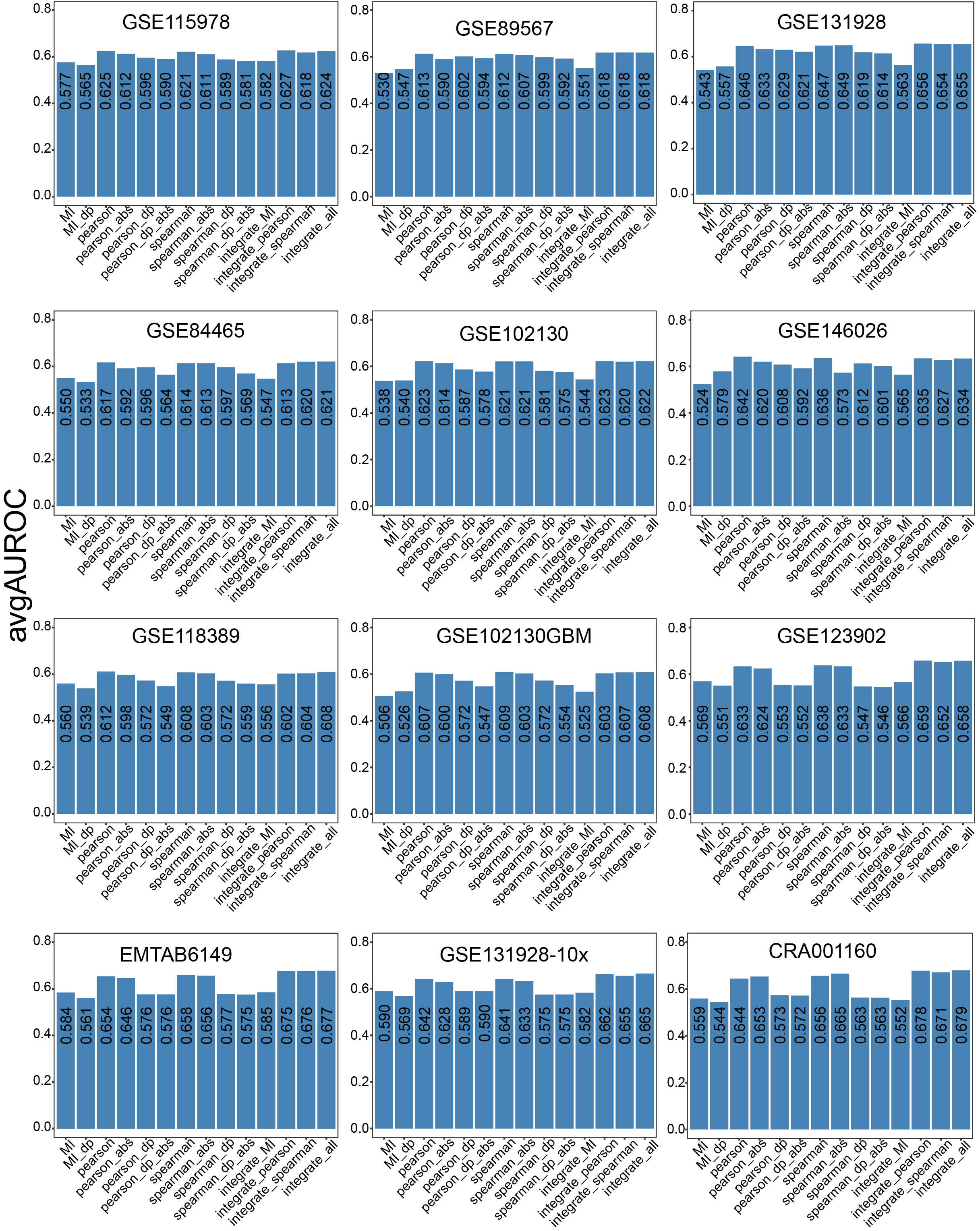


**Figure S20.** The avgAUROC values of gene function prediction based on different single-cell co-expression networks.

## 1.2 Supplementary Tables

**Table S1.** Information about the 14 scRNA-seq datasets.

| **Accession** | **Dataset** | **Cancer type** | **Cell** | **Tumor cell** | **Immune cell** | **Sample** | **Fresh or Frozen** | **Platform** |
| --- | --- | --- | --- | --- | --- | --- | --- | --- |
| GSE131928[1] | GSE131928 | Glioblastoma | 7,858 | 6,801 | 840 | 28 | Fresh | Smart-seq2 |
| GSE115978[2] | GSE115978 | Melanoma | 6,785 | 1,910 | 4,443 | 32 | Fresh | Smart-seq2 |
| GSE89567[3] | GSE89567 | Astrocytoma | 6,341 | 5,097 | 1,048 | 10 | Fresh | Smart-seq2 |
| GSE103322[4] | GSE103322 | Head and neck cancer | 5,016 | 2,195 | 1,090 | 24 | Fresh | Smart-seq2 |
| GSE84465[5] | GSE84465 | Glioblastoma | 2,806 | 877 | 1,639 | 8 | Fresh | Smart-seq2 |
| GSE102130[6] | GSE102130 | H3K27M glioma | 2,596 | 2,259 | 96 | 6 | Fresh | Smart-seq2 |
| GSE146026[7] | GSE146026 | Ovarian cancer | 1,297 | 1,160 | 30 | 9 | Fresh | Smart-seq2 |
| GSE118389[8] | GSE118389 | Breast cancer | 719 | 475 | 104 | 6 | Fresh | Smart-seq2 |
| GSE102130[6] | GSE102130GBM | Glioblastoma | 599 | / | / | 3 | Fresh | Smart-seq2 |
| CRA001160[9] | CRA001160 | Pancreatic ductal adenocarcinoma | 39,607 | 14,314 | 8,702 | 24 | Fresh | 10x Genomics |
| GSE123902[10] | GSE123902 | Lung adenocarcinoma | 13,901 | / | / | 13 | Fresh | 10x Genomics |
| E-MTAB-6149 [11] | EMTAB6149 | Non-small cell lung cancer | 11,493 | 3,718 | 6,106 | 15 | Fresh | 10x Genomics |
| GSE131928[1] | GSE131928-10x | Glioblastoma | 10,983 | / | / | 9 | Fresh | 10x Genomics |
| GSE146026[7] | GSE146026-10x | Ovarian cancer | 6,813 | 2,883 | 2,862 | 8 | Fresh | 10x Genomics |

**Table S2.** Information about the 9 bulk RNA-seq datasets from TCGA.

| **Cancer type** | **Abbreviations** | **Sample** |
| --- | --- | --- |
| Brain Lower Grade Glioma | LGG | 534 |
| Breast invasive carcinoma | BRCA | 1,142 |
| Glioblastoma multiforme | GBM | 169 |
| Head and Neck squamous cell carcinoma | HNSC | 522 |
| Lung adenocarcinoma | LUAD | 517 |
| Non-small cell lung cancer | NSCLC | 1,020 |
| Ovarian serous cystadenocarcinoma | OV | 430 |
| Pancreatic adenocarcinoma | PAAD | 179 |
| Skin Cutaneous Melanoma | SKCM | 472 |

**Table S3.** The threshold values of relevance scores for gene function prediction.

| **Data** | **Threshold value** |  | **Data** | **Threshold value** |
| --- | --- | --- | --- | --- |
| CRA001160 | 0.18 |  | GSE115978 | 0.19 |
| PAAD | 0.17 |  | SKCM | 0.17 |
| T1 | 0.19 |  | Mel04.3 | 0.18 |
| T10 | 0.18 |  | Mel102 | 0.19 |
| T11 | 0.18 |  | Mel103 | 0.19 |
| T12 | 0.2 |  | Mel105 | 0.19 |
| T13 | 0.18 |  | Mel106 | 0.19 |
| T14 | 0.18 |  | Mel110 | 0.19 |
| T15 | 0.19 |  | Mel112 | 0.19 |
| T16 | 0.18 |  | Mel116 | 0.2 |
| T17 | 0.18 |  | Mel121.1 | 0.19 |
| T18 | 0.18 |  | Mel126 | 0.19 |
| T19 | 0.2 |  | Mel128 | 0.19 |
| T2 | 0.18 |  | Mel129pa | 0.19 |
| T20 | 0.18 |  | Mel129pb | 0.19 |
| T21 | 0.18 |  | Mel194 | 0.19 |
| T22 | 0.18 |  | Mel478 | 0.2 |
| T23 | 0.18 |  | Mel53 | 0.18 |
| T24 | 0.18 |  | Mel58 | 0.19 |
| T3 | 0.18 |  | Mel60 | 0.19 |
| T4 | 0.18 |  | Mel71 | 0.19 |
| T5 | 0.18 |  | Mel72 | 0.2 |
| T6 | 0.18 |  | Mel74 | 0.2 |
| T7 | 0.18 |  | Mel75 | 0.19 |
| T8 | 0.18 |  | Mel78 | 0.19 |
| T9 | 0.18 |  | Mel79 | 0.2 |
|  |  |  | Mel80 | 0.19 |
|  |  |  | Mel81 | 0.19 |
|  |  |  | Mel82 | 0.19 |
|  |  |  | Mel84 | 0.19 |
|  |  |  | Mel88 | 0.19 |
|  |  |  | Mel89 | 0.2 |
|  |  |  | Mel94 | 0.19 |
|  |  |  | Mel98 | 0.19 |

# References

1. Neftel, C.; Laffy, J.; Filbin, M.G.; Hara, T.; Shore, M.E.; Rahme, G.J.; Richman, A.R.; Silverbush, D.; Shaw, M.L.; Hebert, C.M.; et al. An Integrative Model of Cellular States, Plasticity, and Genetics for Glioblastoma. *Cell* **2019**, *178*, 835-849 e821, doi:10.1016/j.cell.2019.06.024.

2. Jerby-Arnon, L.; Shah, P.; Cuoco, M.S.; Rodman, C.; Su, M.J.; Melms, J.C.; Leeson, R.; Kanodia, A.; Mei, S.; Lin, J.R.; et al. A Cancer Cell Program Promotes T Cell Exclusion and Resistance to Checkpoint Blockade. *Cell* **2018**, *175*, 984-997 e924, doi:10.1016/j.cell.2018.09.006.

3. Venteicher, A.S.; Tirosh, I.; Hebert, C.; Yizhak, K.; Neftel, C.; Filbin, M.G.; Hovestadt, V.; Escalante, L.E.; Shaw, M.L.; Rodman, C.; et al. Decoupling genetics, lineages, and microenvironment in IDH-mutant gliomas by single-cell RNA-seq. *Science* **2017**, *355*, doi:10.1126/science.aai8478.

4. Puram, S.V.; Tirosh, I.; Parikh, A.S.; Patel, A.P.; Yizhak, K.; Gillespie, S.; Rodman, C.; Luo, C.L.; Mroz, E.A.; Emerick, K.S.; et al. Single-Cell Transcriptomic Analysis of Primary and Metastatic Tumor Ecosystems in Head and Neck Cancer. *Cell* **2017**, *171*, 1611-1624 e1624, doi:10.1016/j.cell.2017.10.044.

5. Darmanis, S.; Sloan, S.A.; Croote, D.; Mignardi, M.; Chernikova, S.; Samghababi, P.; Zhang, Y.; Neff, N.; Kowarsky, M.; Caneda, C.; et al. Single-Cell RNA-Seq Analysis of Infiltrating Neoplastic Cells at the Migrating Front of Human Glioblastoma. *Cell Rep* **2017**, *21*, 1399-1410, doi:10.1016/j.celrep.2017.10.030.

6. Filbin, M.G.; Tirosh, I.; Hovestadt, V.; Shaw, M.L.; Escalante, L.E.; Mathewson, N.D.; Neftel, C.; Frank, N.; Pelton, K.; Hebert, C.M.; et al. Developmental and oncogenic programs in H3K27M gliomas dissected by single-cell RNA-seq. *Science* **2018**, *360*, 331-335, doi:10.1126/science.aao4750.

7. Izar, B.; Tirosh, I.; Stover, E.H.; Wakiro, I.; Cuoco, M.S.; Alter, I.; Rodman, C.; Leeson, R.; Su, M.J.; Shah, P.; et al. A single-cell landscape of high-grade serous ovarian cancer. *Nat Med* **2020**, *26*, 1271-1279, doi:10.1038/s41591-020-0926-0.

8. Karaayvaz, M.; Cristea, S.; Gillespie, S.M.; Patel, A.P.; Mylvaganam, R.; Luo, C.C.; Specht, M.C.; Bernstein, B.E.; Michor, F.; Ellisen, L.W. Unravelling subclonal heterogeneity and aggressive disease states in TNBC through single-cell RNA-seq. *Nature communications* **2018**, *9*, 3588, doi:10.1038/s41467-018-06052-0.

9. Peng, J.; Sun, B.F.; Chen, C.Y.; Zhou, J.Y.; Chen, Y.S.; Chen, H.; Liu, L.; Huang, D.; Jiang, J.; Cui, G.S.; et al. Single-cell RNA-seq highlights intra-tumoral heterogeneity and malignant progression in pancreatic ductal adenocarcinoma. *Cell Res* **2019**, *29*, 725-738, doi:10.1038/s41422-019-0195-y.

10. Laughney, A.M.; Hu, J.; Campbell, N.R.; Bakhoum, S.F.; Setty, M.; Lavallee, V.P.; Xie, Y.; Masilionis, I.; Carr, A.J.; Kottapalli, S.; et al. Regenerative lineages and immune-mediated pruning in lung cancer metastasis. *Nat Med* **2020**, *26*, 259-269, doi:10.1038/s41591-019-0750-6.

11. Lambrechts, D.; Wauters, E.; Boeckx, B.; Aibar, S.; Nittner, D.; Burton, O.; Bassez, A.; Decaluwe, H.; Pircher, A.; Van den Eynde, K.; et al. Phenotype molding of stromal cells in the lung tumor microenvironment. *Nat Med* **2018**, *24*, 1277-1289, doi:10.1038/s41591-018-0096-5.
